# Genetic discovery for oil production and quality in sesame

**DOI:** 10.1038/ncomms9609

**Published:** 2015-10-19

**Authors:** Xin Wei, Kunyan Liu, Yanxin Zhang, Qi Feng, Linhai Wang, Yan Zhao, Donghua Li, Qiang Zhao, Xiaodong Zhu, Xiaofeng Zhu, Wenjun Li, Danlin Fan, Yuan Gao, Yiqi Lu, Xianmei Zhang, Xiumei Tang, Congcong Zhou, Chuanrang Zhu, Lifeng Liu, Ruichun Zhong, Qilin Tian, Ziruo Wen, Qijun Weng, Bin Han, Xuehui Huang, Xiurong Zhang

**Affiliations:** 1 Key Laboratory of Biology and Genetic Improvement of Oil Crops, Ministry of Agriculture of People's Republic of China, Oilcrops Research Institute, Chinese Academy of Agricultural Sciences, Wuhan 430062, China; 2 National Center for Gene Research, Collaborative Innovation Center for Genetics and Development, Institute of Plant Physiology and Ecology, Shanghai Institutes for Biological Sciences, Chinese Academy of Sciences, 500 Caobao Road, Shanghai 200233, China; 3 Luohe Academy of Agricultural Sciences, Luohe 462300, China; 4 Cash Crops Research Institute, Guangxi Academy of Agricultural Sciences, Nanning 530007, China

## Abstract

Oilseed crops are used to produce vegetable oil. Sesame (*Sesamum indicum*), an oilseed crop grown worldwide, has high oil content and a small diploid genome, but the genetic basis of oil production and quality is unclear. Here we sequence 705 diverse sesame varieties to construct a haplotype map of the sesame genome and *de novo* assemble two representative varieties to identify sequence variations. We investigate 56 agronomic traits in four environments and identify 549 associated loci. Examination of the major loci identifies 46 candidate causative genes, including genes related to oil content, fatty acid biosynthesis and yield. Several of the candidate genes for oil content encode enzymes involved in oil metabolism. Two major genes associated with lignification and black pigmentation in the seed coat are also associated with large variation in oil content. These findings may inform breeding and improvement strategies for a broad range of oilseed crops.

## Abstract (web summary)

Sesame is a valuable oilseed crop with a small diploid genome and high seed-oil content making it an attractive model for genetic studies. Here, Wei *et al.* sequence more than 705 sesame varieties and perform a genome-wide association study to identify genes associated with important agronomic traits.

## Graphical abstract

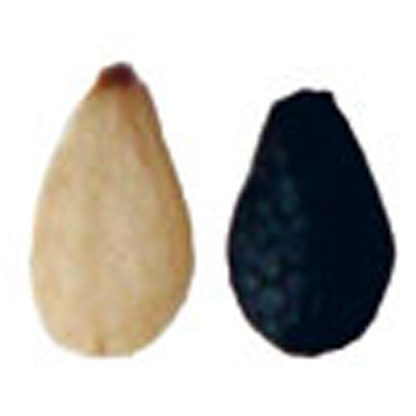

Humans rely on agricultural crops that contain an abundance of starches, oils and proteins, to obtain food and fodder. Among the grasses (monocots), many cereal crops (for example, corn, wheat, rice, barley and sorghum) have been successfully domesticated for producing numerous starch-rich grain seeds. Oilseed crops, which are primarily grown for the oil found in the seeds are mostly dicots, including rapeseed, peanut, soybean, sunflower and sesame (*S. indicum*)^1^. Compared with cereal crops, genetic investigation of oilseed crops has been limited^2^,^3^,^4^,^5^,^6^, although the demand for vegetable oil is increasing worldwide. Among the oilseed crops, sesame has high oil content (∼55% in the seeds) and a small diploid genome (∼350 Mb)^7^, making it an attractive species for genetic studies. Sesame oil has been suggested to have health benefits and is very popular in many countries^8^. As one of the oldest crops, sesame is widely cultivated in many tropical and subtropical regions^9^,^10^. Under long-term natural and artificial selection coupled with a wide geographic distribution, sesame has a large number of diverse varieties, which can be important resources for genetic investigation and breeding.

Genome-wide association study (GWAS) can use the diverse varieties to take full advantage of ancient recombination events and identify the loci underlying complex traits at relatively high resolution. GWAS is a well-established methodology in plant genetics, owing to the development of sequencing technologies and coupled computation methods. These developments greatly accelerate the construction of high-density haplotype maps, which comprehensively capture the genomic variation and the pattern of common haplotypes within the species. Up to now, GWAS have been successfully applied in many plant species (for example, *Arabidopsis*, rice, maize, foxtail millet and soybean)^11^,^12^,^13^,^14^,^15^,^16^,^17^. To dissect the genetic architecture of oil-related traits in maize, 368 inbred lines were analysed at ∼1 million single-nucleotide polymorphisms (SNPs) and 74 loci were found to be associated with maize kernel oil concentration and fatty acid composition^16^.

The completion of a high-quality reference genome sequence in sesame provides an opportunity to fully investigate the genetic architecture of oil-related traits in this typical oilseed crop^7^. In this study, we collected and sequenced a large number of cultivated sesame varieties, aiming to extend our understanding of storage-oil regulation and oilseed yield. By exploiting the natural variation in these 705 sesame genomes and performing a large-scale GWAS on 56 agronomic traits, key genomic loci underlying oil content, nutritional quality and oilseed yield of sesame were systemically identified for the first time. Moreover, forty-six candidate causative genes were identified by integrating functional genomic information. This genetic resource may potentially be used to further breeding and biotechnology-assisted improvement of sesame and other oilseed crops.

## Results

### Genomic variation from population-scale sequencing

To enable large-scale genetic analysis, we first constructed a haplotype map of 705 diverse sesame accessions that were collected across the world, covering all the major planting areas of sesame (Supplementary Data 1). We sequenced these sesame accessions using the Illumina HiSeq2500 system, each with ∼2.6-fold genome coverage (Supplementary Fig. 1), generating a total of 0.6 trillion base pairs (bp) of raw data (96-bp paired-end reads). The sesame reference genome sequence contains 27,148 annotated genes and has a relatively low proportion of repetitive sequences (28.5%) (ref. 7). We aligned the reads against the sesame reference genome sequence and only the uniquely mapped reads (70.2% of raw reads) in the reference genome (in total 1,269-fold read depth) were used for SNP calling. A total of 5,407,981 SNPs were identified in the sesame genome with an average of 1 SNP per 50 bp. We investigated the potential effects of the SNPs in coding regions and identified a total of 254,781 non-synonymous SNPs in 24,089 genes (96.8% of all the sesame genes), which included 11,041 large-effect SNPs that caused premature stop codon or start codon changes in 7,801 genes.

Moreover, we sequenced two genetically distinct sesame varieties Baizhima and Mishuozhima with ∼70 × genome coverage. The two genomes were further *de novo* assembled and the final assemblies had a contig N50 size of ∼47 kb (Supplementary Tables 1 and 2). The protein-coding genes were annotated for each genome and all the sequence variants were detected through alignment with the sesame reference genome^7^. Large-effect variants (including insertions or deletions (indels) leading to frameshifts in gene coding sequences) were found in 2,673 genes including some well-characterized genes. For example, a 1-bp deletion was detected in the coding regions of *SiGI* (SIN_1015799, an orthologue of *GIGANTEA*, which is involved in photoperiod-mediated flowering in *Arabidopsis*^18^) in the genome of the Baizhima variety (Supplementary Fig. 2).

### Large-scale genetic discovery of 56 traits

**Figure 1:**
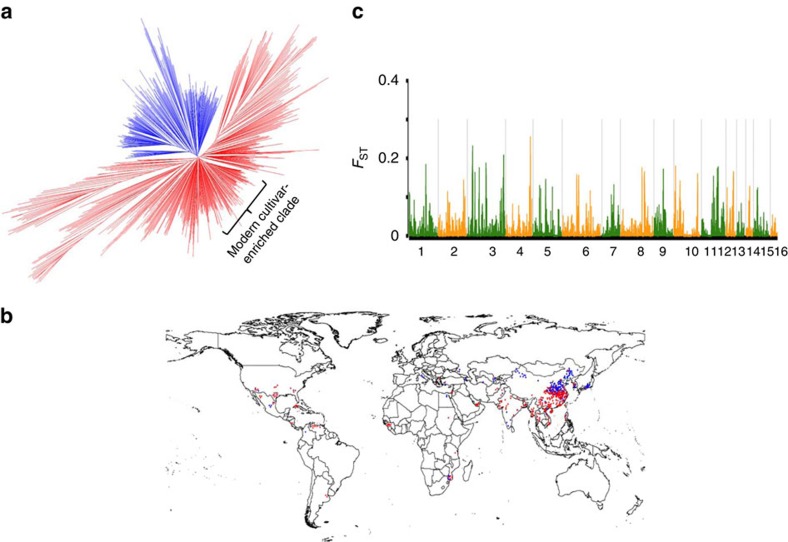
Phylogenetic tree and geographic distributions of 705 sesame varieties. (**a**) Neighbour-joining tree of all the varieties calculated from whole-genome SNPs. The two recognizable groups are coloured in red and blue, respectively. (**b**) Geographic distributions of all the varieties are indicated as spots in the world map, with the two groups colour coded as in **a**. (**c**) The level of genetic differentiation (*F*_ST_) between the two groups is plotted against the whole genome.

We explored the population structure of the 705 sesame accessions (Supplementary Fig. 3) and found that the structure generally correlated with the latitude distribution of the accessions (Pearson's correlation *r*^*2*^=0.25 between the first principal component and latitude, *P*<0.0001, Pearson's correlation test). The neighbour-joining tree identified two recognizable groups, one of which tends to be from northern areas (coloured in blue in Fig. 1a,b). Sesame is thought to have originated in South Asia^19^,^20^ and the northern-area group may be derived from the long-term selection for adaptation to the photoperiod and temperature changes. Nevertheless, genome-wide genetic differentiation between the two groups was very weak, with an average *F*_st_ index of 0.02 (0.020 and 0.022 with and without the modern cultivars, respectively; Fig. 1c). The sesame germplasm collection included 95 modern cultivars developed during recent decades. The modern cultivars showed lower nucleotide diversity than landraces and were enriched in a small clade of the phylogenetic tree (Fig. 1a and Supplementary Fig. 4). The low population differentiation, coupled with a modest level of nucleotide diversity (2.4 × 10^−3^) and a modest level of LD decay rate (the average pairwise correlation coefficient dropped to half at 88 kb from the initial value 0.55; Supplementary Fig. 5), are advantageous for GWAS in sesame.

**Figure 2:**
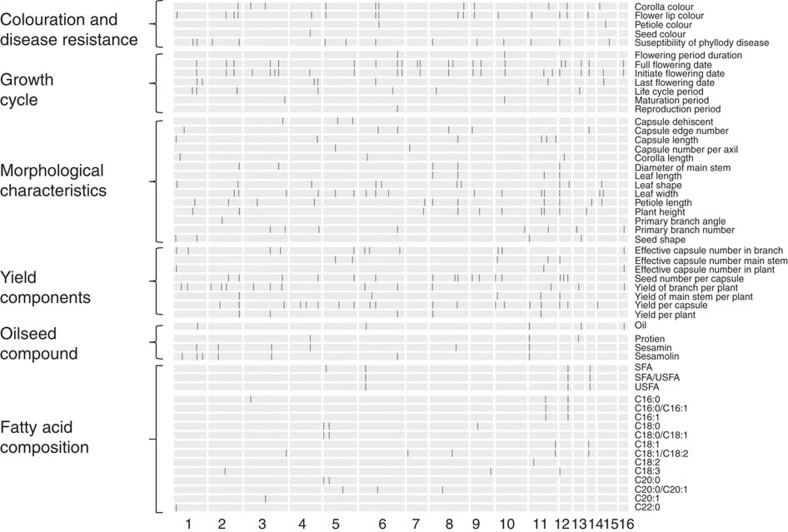
Large-scale genetic discoveries of agronomic traits in sesame. For each trait, the associated loci (*P*<1 × 10^−6^) are indicated in the genome. The agronomic traits (labelled on the right) can be divided into six categories (labelled on the left). Among them, the traits of fatty acid composition were measured in Luohe and all the others were phenotyped in Sanya.

The 705 sesame accessions were planted in four agro-ecologically diverse locations in China, for extensive phenotyping (Supplementary Fig. 6 and Supplementary Data 2). A total of 56 agronomically important traits were measured, including traits involved in the oil content, nutrient composition, yield components, morphological characteristics, growth cycle, colouration and disease resistance. Most traits had abundant phenotypic diversity (Supplementary Fig. 7). We performed a large-scale GWAS study on a total of 169 sets of phenotypic data using the genotypic data from 1,805,413 common SNPs with minor allele frequency (MAF) >0.03. The genotype data set contained a number of missing calls, which were imputed using *k*-nearest neighbour algorithm^12^. We randomly selected ten sesame accessions for additional high-coverage sequencing and independent genotype calling. We compared the imputed genotypes with those acquired from high-coverage sequencing and the overall concordance was 97.8% (Supplementary Table 3). We identified 549 peaks that were associated with the phenotypic variation above a suggestive threshold (*P*<1 × 10^−6^ in mixed model, false discovery rate (FDR) <0.05), of which 303 peaks could exceed a stricter cutoff (*P*<1 × 10^−7^ in mixed model, after Bonferroni correction, FDR <0.01; Fig. 2, Supplementary Figs 8–11 and Supplementary Data 3). There were 446 significant associations with >5% MAF and 103 associations with low frequency (3%–5% MAF). The full lists of all the significant associations are presented in Supplementary Data 3. According to the distribution of the association signals in sesame genome (Supplementary Fig. 12), we identified 17 hotspots with significantly more association signals than expected (*P*<0.01, binomial test).

Some traits were controlled by one or two major loci that explained a large proportion of the phenotypic variation, such as flower lip and petiole colour (Supplementary Figs 13 and 14). The major loci were subsequently examined through in-depth analyses, to pinpoint the candidate causative genes. Candidate genes were selected in the associated loci if they encode components of metabolic or signalling pathways known to be related to the corresponding phenotypes or based on expression profile (for example, tissue-specific expression) using RNA sequencing (RNA-seq) data from multiple tissues in sesame. For example, we identified *SiGL3* as a candidate gene for flower lip colour, as it is predicted to encode a component of the anthocyanin biosynthetic pathway^21^. Likewise, *SiMYB113* and *SiMYB23* were considered candidate genes for petiole colour, as they are homologous to transcription factors that regulate anthocyanin biosynthesis in other species^22^. Several candidate genes for flowering time divergence were homologues of known photoperiod genes (*SiCOL5*, *SiELF9*, *SiGF14*, *SiGI* and *SiTOC1*)^18^,^23^,^24^,^25^,^26^ and several genes encoding nucleotide binding site-leucine-rich repeats (NBS-LRR) were considered candidates for variation in disease resistance given the role of homologues in other species^27^. In total, 46 candidate causative genes were identified (Supplementary Data 4). Homologues of some of these genes, especially those for fatty acid composition, had been studied in other species such as *Arabidopsis*^28^ and maize^16^ (Supplementary Data 5).

### Major genes for oil content and composition

**Figure 3:**
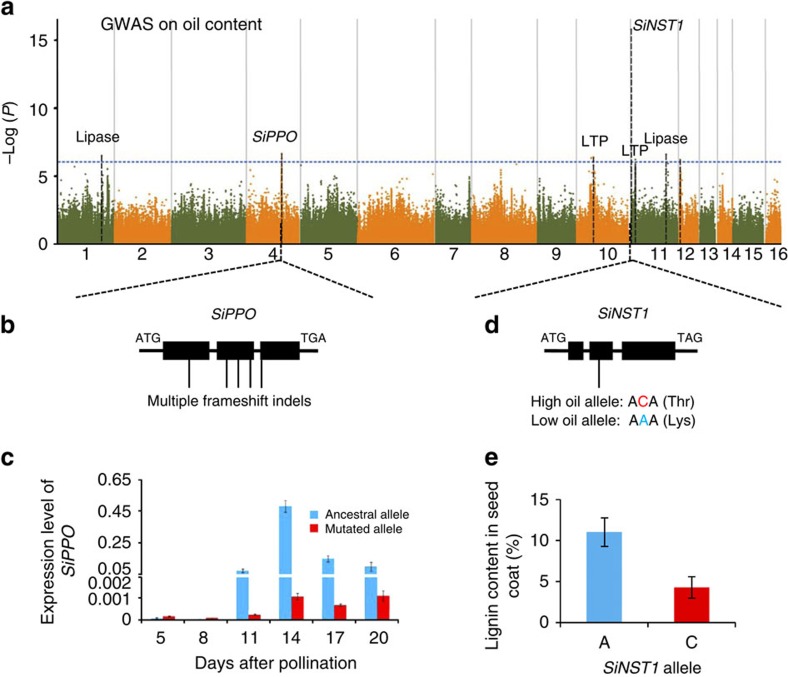
Candidate causative genes and variants underlying oil content in sesame oilseeds. (**a**) Negative log_10_ *P*-values for association of oil content in Luohe (*Y* axis) are plotted against SNP positions (*X* axis). The genome-wide significant *P*-value threshold (10^−6^) is indicated by a horizontal dash–dot line. The candidate genes are indicated near the association peaks. (**b**) Candidate causative variants in *SiPPO*. (**c**) qRT–PCR result of *SiPPO* in seeds. The mutated allele (with high oil content) shows quite low transcripts. The bar indicates s.d. (**d**) The candidate causative variants (a C-to-A missense SNP) in *SiNST1*. (**e**) The content of lignin in the seed coats from 14 sesame accessions. The varieties with ‘A' allele show higher level of lignin than those with ‘C' allele significantly (*P*<0.0001, Student's *t*-test). The bar indicates s.d.

Among the seeds of the 705 sesame varieties, the oil content (the weight of all oil compounds per unit weight of intact seeds) varied from 40.83% to 61.88%. We identified a total of 13 significant associations (*P*<1 × 10^−6^ in mixed model). Seven of them were identified in the phenotyping location Luohe and together explained 44.4% of the variation in oil content (Fig. 3a). For each of the associations, we examined whether the high-oil alleles had negative effects on the seed yield. Fortunately, there were no significant associations observed between the allelic variation for the seed oil content and the yield traits, and the phenotypic correlation between oil content and oilseed yield is also weak (Pearson's correlation *r*^2^=0.02), suggesting that it would be possible to generate sesame varieties with both high yield and high oil content.

Among the seven associations (Fig. 3a), four loci contained genes encoding components of the oil metabolic pathway, including two genes encoding lipases (*CXE17*, SIN_1003248 and GDSL-like lipase, SIN_1013005) and two encoding lipid transfer proteins (SIN_1019167 and SIN_1009923). The candidate causative genes at the other two loci (*SiPPO* and *SiNST1*) are not predicted to have direct involvement in oil biosynthesis (Fig. 3b–e). For the remaining locus, no strong evidence for a candidate causal gene could be found.

The gene with the strongest association for oil content (*P*=1.70 × 10^−16^ in mixed model) in sesame also had the strongest association for sesamin, sesamolin (two lignan compounds, which are beneficial to human health^29^,^30^) and protein content in sesame seeds. The strongest association signal was a missense SNP (from C to A) located within *SiNST1* (SIN_1005755) that results in a change from T to K at the 82nd amino acid position (Fig. 3d). Sequence comparison between Zhongzhi13 (high-oil allele with ‘C') and Mishuozhima (low-oil allele with ‘A') indicated that this missense SNP was the only coding region variant around the local 100-kb genomic region. The quantitative reverse-transcriptase PCR (qRT–PCR) results revealed that *SiNST1* has a very high expression level in seeds 8 days after pollination (DAP) of the ‘A' haplotype and 14 DAP of the ‘C' haplotype, and *SiNST1* has relatively low expression in stems and roots at vegetative period and very low expression in flowers and leaves (Supplementary Fig. 15). In sesame seeds, the ‘A' allele of *SiNST1* (MAF=9%) was associated with significantly decreased content of oil, protein, sesamin and sesamolin, but increased content of lignin (*P*<0.0001, *t*-test; Fig. 3e) and seed coat thickness (*P*<0.01, *t*-test; Supplementary Fig. 15), which is consistent with the function of its *Arabidopsis* orthologue. *AtNST1* is reported to regulate secondary wall formation, lignin biosynthesis and cellulose content in woody tissues^31^,^32^. We propose that the ‘A' allele of *SiNST1* probably promotes the accumulation of woody tissues in seeds, thereby decreasing the content of other nutrients (for example, oil and protein) in the whole seed and could also affect the biosynthesis of other components. Validation of the causal polymorphisms will require further functional analysis.

Another major locus that we discovered for the oil content contained the candidate gene *SiPPO* (SIN_1016759). Sequence comparison revealed multiple nonsense SNPs and frameshift indels in *SiPPO* (Fig. 3b). We noticed that 98 of 404 landraces and 2 of 95 modern cultivars harbour the ancestral alleles, respectively (showing significant divergence, *P*=2 × 10^−6^, *χ*^2^-test). Population genetics analysis suggested that *SiPPO* was under selection in modern breeding (*π*_landrace_/*π*_cultivar_=3.71 versus the genome-wide level 1.37), probably due to the mutated *SiPPO* playing a role in increasing oil content. Based on qRT–PCR and RNA-seq data, *SiPPO* had almost no expression in varieties with the mutated *SiPPO* allele (Fig. 3c and Supplementary Fig. 16). In the varieties with the ancestral allele, the gene was highly expressed in the seeds from 11 to 20 DAP. Notably, *SiPPO* was also strongly associated with seed coat colour (*P*=9.33 × 10^−130^ in mixed model) and seed protein content (*P*=1.02 × 10^−7^ in mixed model; Supplementary Fig. 16). *SiPPO* encodes a predicted polyphenol oxidase. This enzyme has been reported to produce black pigments through the browning reaction^33^,^34^ and is thereby probably responsible for generating black sesame (with the ancestral allele) or white sesame (with the mutated allele). It is unclear how pigmentation is related to oil biosynthesis.

**Figure 4:**
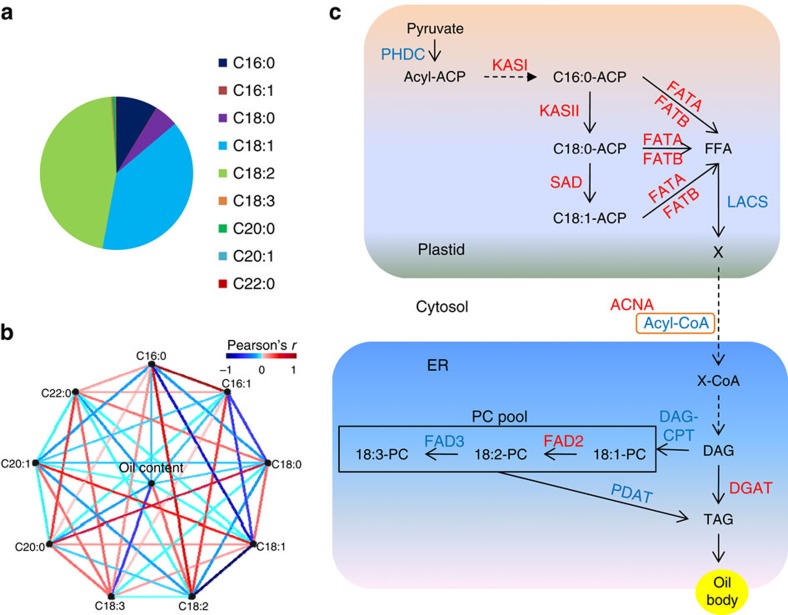
Network of genes controlling oil composition. (**a**) Fatty acid composition in sesame seed. (**b**) Correlation of phenotypic variation for oil content and composition. (**c**) The simplified lipid metabolic pathway. The pathway is modified from *Arabidopsis* and maize^16^,^28^. The candidate causative genes discovered for the variation of fatty acid synthesis in sesame germplasm are highlighted in red. The dotted lines represent multiple reaction steps. The plastid, endoplasmic reticulum (ER) and oil body are marked with light orange, light blue and yellow, respectively.

Oilseed quality includes both seed oil content and fatty acid composition. The concentration of 11 fatty acids was measured in this study and 40 association peaks were identified. Among the fatty acids, palmitic acid (C16:0, ranging from 6.92% to 11.16% in the varieties), stearic acid (C18:0, 3.97% to 5.97%), oleic acid (C18:1, 32.08% to 53.14%) and linoleic acid (C18:2, 32.95% to 52.49%) accounted for a predominant proportion of the oil content (Fig. 4a). The content of different fatty acids was often correlated and shared associations with common genetic loci (Fig. 4b and Supplementary Table 4). For example, the candidate causative genes for two highly correlated traits, the palmitic acid (C16:0) concentration and palmitoleic acid (C16:1) concentration were the same—*SiKASI* (SIN_1001803) and *SiKASII* (SIN_1024652) (Supplementary Fig. 17). The candidate genes underlying the variation in the fatty acid composition of sesame varieties also included *SiACNA*, *SiDGAT2*, *SiFATA*, *SiFATB* and *SiSAD*. Homologues of most of these genes are involved in lipid metabolism in the plastid and endoplasmic reticulum, in particular in fatty acid elongation, desaturation and export from plastid^28^ (Fig. 4c and Supplementary Data 5). Notably, there were only weak correlations between oil content and composition of all fatty acids (Supplementary Table 4). Accordingly, there were no overlaps between the associated loci for oil content and those for oil composition.

The unsaturated to saturated fat ratio is an important index for evaluating the quality of edible oil. We found that sesame oil contained a stably high proportion of unsaturated fatty acid, ranging from 83.40% to 86.97%. We found that *SiKASI* and *SiDGAT2* were the major genes associated with the unsaturated to saturated fat ratio with significant association signals (*P*=1.45 × 10^−16^ and *P*=2.57 × 10^−7^, respectively). *SiDGAT2* is homologous to a gene encoding a key enzyme required for triacylglycerol synthesis^35^, whereas homologues of *SiKASI* encode enzymes involved in palmitic acid synthesis^28^. There was no sequence variation occurred in the coding region of *SiKASI* and the causative variant was probably located in the promoter region (Supplementary Fig. 18). The *SiFAD2* gene (SIN_1009785) is predicted to encode an oleic acid desaturase, which have been reported to act as a key node for converting oleic acid to linoleic acid in the endoplasmic reticulum^36^. Whether and how *SiFAD2* contributes to the natural genetic variation in the level of oleic acid in sesame remain unknown. The GWAS study on oleic acid composition showed that the association signal at the locus was modest (*P*=2.3 × 10^−5^ in mixed model) but did not pass the genome-wide threshold. The coding variants in *SiFAD2* in the 705 accessions were then specifically screened. Varieties with a missense mutation (R to H at the 142nd amino acid, which probably affects the desaturase activity) all had an extremely high content of oleic acid in seed oil (Supplementary Fig. 19). However, the coding SNP has a very low MAF (1.5%), which was thereby not included in the whole-genome screening.

### Genetic architecture of oilseed yield traits

**Figure 5:**
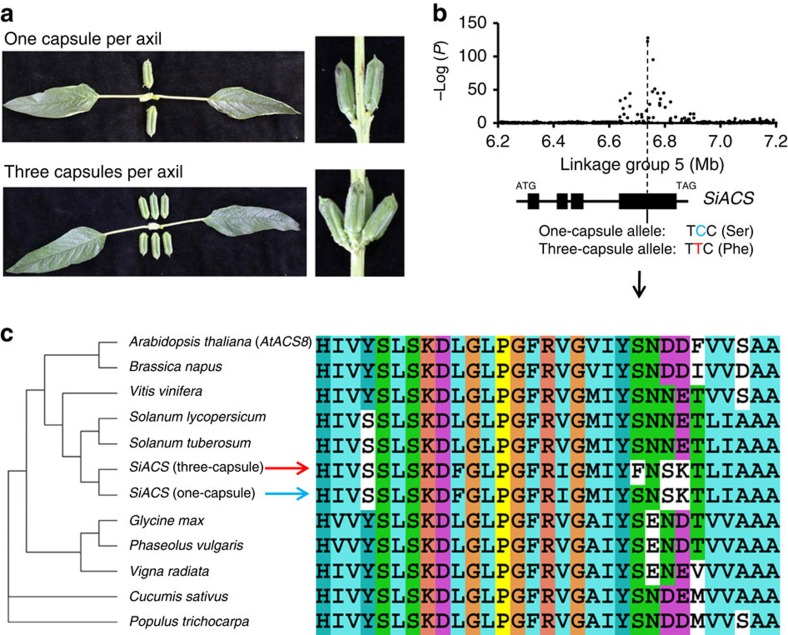
A major gene for oilseed yield in sesame breeding. (**a**) Photos of the phenotypic change from one capsule per axil in wild sesame to three capsules per axil in modern cultivars. (**b**) Negative log_10_ *P*-values for association of capsule number per axil in Sanya (*Y* axis) are plotted against SNP positions (*X* axis). The association peak is indicated. (**c**) The homologues of *SiACS* in plants and local alignment of protein sequences of the homologues around the candidate causative variant.

For improvement of oilseed crops, breeders pay attention not only to oil quality (for example, changes in fatty acid composition) but also the oilseed yield. An interesting improvement in the seed yield of sesame associated with domestication is the alteration of the capsule number (the number of fruits that store oilseeds), from one capsule per axil in wild sesame to three capsules in many modern cultivars (Fig. 5a). The GWAS study result demonstrated that this significant change was primarily associated with a single locus that could explain up to 60% of the phenotypic variation (*P*=1.02 × 10^−128^ in mixed model). The peak signal of the locus was a missense SNP located within *SiACS* (SIN_1006338) that led to F/S variation at the 284th amino acid of the protein. This missense SNP was the only coding variant around the local 100-kb region based on a comparison between Zhongzhi13 (three-capsule allele) and Baizhima (one-capsule allele), suggesting that *SiACS* is probably the causative gene (Fig. 5b). The predicted one-capsule allele (encoding the amino acid F) is the ancestral type according to the information from wild sesame and its homologues in other plants (Fig. 5c). Moreover, this allele was completely dominant through an analysis of a biparental population (a cross between Zhongzhi13 and Baizhima). The homologue of *SiACS* in *Arabidopsis* (*AtACS8*) was reported to be an auxin-induced gene involved in ethylene biosynthesis^37^, suggesting that the one/three-capsule phenotype may be under the regulation of plant hormones. We observed that the *SiACS* locus had a large effect on many other traits, including the leaf width and yield of the main stem. The varieties with the predicted three-capsule allele have a significantly wider leaf than those with the one-capsule allele (*P*=5.75 × 10^−11^ in mixed model), suggesting that the source–sink relationship in plants may be an important contributor to seed yield.

**Figure 6:**
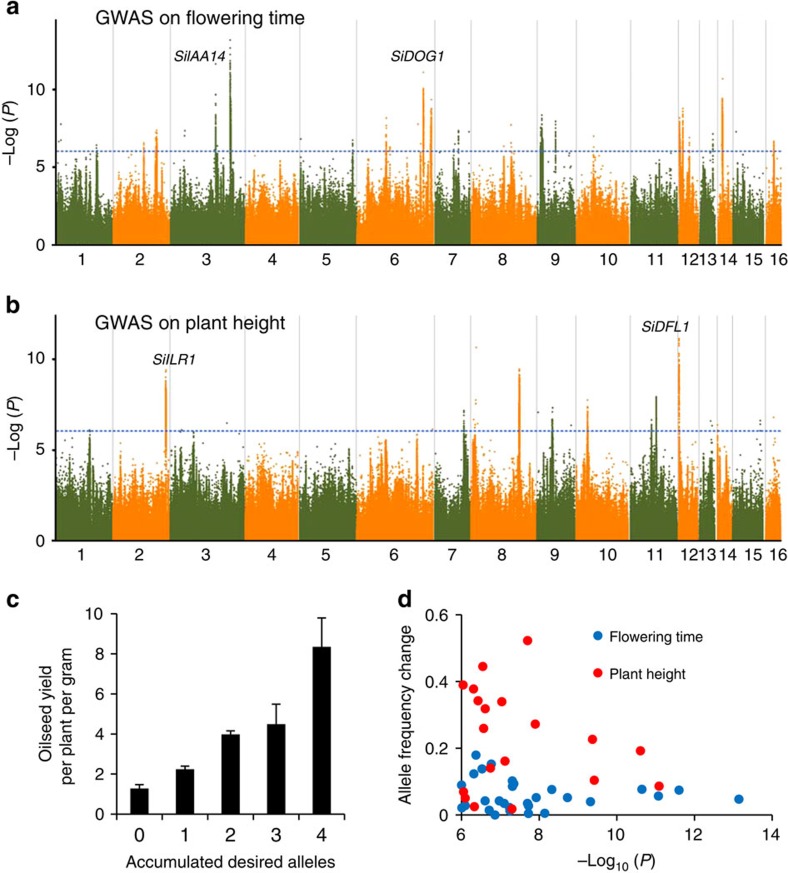
Genetic analysis of flowering time and plant height for oilseed yield. (**a**) Negative log_10_ *P*-values for association of flowering time in Sanya (*Y* axis) are plotted against SNP positions (*X* axis). Two loci (*SiDOG1* and *SiIAA14*) significantly associated with oilseed yield are indicated. (**b**) Negative log_10_ *P*-values for association of plant height in Sanya (*Y* axis) are plotted against SNP positions (*X* axis). Two loci (*SiDFL1* and *SiILR1*) significantly associated with oilseed yield are indicated. (**c**) Pyramiding of desired alleles (at *SiDOG1*, *SiIAA14*, *SiDFL1* and *SiILR1*) in sesame varieties. The varieties accumulated the desired alleles generally shows better yield performance. The bar indicates s.e. (**d**) Allele frequency changes from landraces to modern cultivars for all lead SNPs underlying flowering time and plant height. The loci for plant height showed to be selected more intensively than those for flowering time.

Other yield-related traits were mostly determined by multiple loci with modest or small genetic effects. Together, 29 peaks could explain 68.0% of the phenotypic variation in flowering time and 19 peaks explained 56.2% of the variation in the plant height. It is noteworthy that these proportions may be partial overestimates due to Beavis effect^38^, because the mean *r*^2^ value on randomized data was calculated to be ∼29.9% for the traits. Two candidate genes at flowering-time loci (*SiDOG1* and *SiIAA14*)^39^,^40^ and two candidate genes at plant-height loci (*SiDFL1* and *SiILR1*)^41^,^42^ were significantly associated with oilseed yield (Fig. 6a,b and Supplementary Data 4). As expected, the alleles with a longer growth stage and larger plant height were coupled with a much higher oilseed yield (Fig. 6c and Supplementary Table 5).

We calculated the allele frequencies of all the trait-associated lead SNPs in landraces and modern cultivars, and found that 31 loci were intensively selected where the allele frequencies between landraces and modern cultivars has an alteration of >30% (Supplementary Data 6). For example, at the *SiACS* locus, the frequency of the predicted three-capsule allele was rapidly increased by recent breeding, from 59.5% in landraces to 98.9% in modern cultivars. The selected alleles often corresponded to a better yield performance, especially for the traits such as capsule number, seed number and plant height (Fig. 6d and Supplementary Fig. 20). However, the selected loci occupied only a small proportion of the yield-related loci that we identified. In fact, the desired alleles at many associated loci underlying yield-related traits have not yet been intensively selected and are far from being fixed, suggesting promising yield potential in the future.

## Discussion

Taken together, our work provides a large data set of genomic variation for diverse varieties and a comprehensive landscape of the important loci and genes for oil traits in the oilseed crop sesame. Previous studies on the mechanisms of lipid biosynthesis and accumulation have identified dozens of lipid-related genes^28^. In the sesame varieties, four loci underlying the natural variation of oil content in seeds pinpointed the candidate genes that were involved in the lipid transfer and lipid hydrolysis pathways. However, the loci underlying oil content in sesame were found to not always be the enzymes in the oil biosynthetic pathways. Sesame seeds also contain high amounts of protein and dietary fibre (including lignin and cellulose). The genes regulating the non-oil components in oilseeds (for example, *SiPPO* for black pigmentation in the seed coat and *SiNST1* involved in lignin and cellulose biosynthesis) may affect the oil content indirectly. The relevant genes and pathways involved in the formation, accumulation and regulation of various compounds in sesame oilseeds have not been fully elucidated. In future works, comprehensive metabolic profiling, coupled with metabolomics GWAS using the well-sequenced varieties, may provide more clues and knowledge regarding the biochemical relevance of important genes in oilseeds.

In sesame oil, fatty acid composition was found to be regulated by a few key members in the oil metabolic pathways including fatty acid elongation, desaturation, export from plastid and the triacylglycerol biosynthesis in the endoplasmic reticulum. Among them, *SiKASI* (3-ketoacyl-acyl carrier protein synthase) in palmitic acid synthesis and *SiDGAT2* (diacylglycerol acyltransferase) for triacylglycerol synthesis were probably the major genes underlying the variation of the unsaturated to saturated fat ratio, an index for healthy dietary consumption. For the unsaturated fat, the proportion of oleic acid is another important index for oil quality. The oleic acid composition in sesame oil was not high (∼39%) when compared with some other crops (for example, 70%–80% in olive oil). A missense mutation in *SiFAD2* and the allelic variation in *SiSAD* (stearoyl-acyl-carrier-protein desaturase) could increase the oleic acid content to ∼48%. To develop sesame cultivars with higher oleic acid proportion, more allelic variation need to be screened and marker-assisted selection of the favourite alleles in multiple gene loci was needed.

The candidates we identified for oil traits, although some of them are highly suggestive, are still putative causative genes. Construction of multiple biparental populations from well-designed crosses will allow the improvement of mapping resolution, the identification of epistatic interactions and the generation of new germplasm with better phenotypic performance. Functional genomics methodologies, such as genetic transformation and genome-editing technologies using CRISPR/Cas system, are much needed to validate the effects of these candidate genes and their functional variants for the associations underlying oil traits. The genes identified in sesame for oil production and quality probably play important roles in other closely related oilseed species (for example, sunflower) as well, offering the opportunity to look for genes with common function. For ongoing efforts in the genetic studies for the oilseed crops with more complex genomes, this work in sesame may provide unique information and guiding examples.

Our GWAS study panel primarily included traditional landraces, coupled with a small number of modern cultivars as well. To understand the genetic transition from a wild plant with low oil content and much low oilseed yield to a typical oilseed crop, diverse *Sesamum malabaricum* accessions (the direct wild progenitor of cultivated sesame, growing in the Indian subcontinent) is under collection for whole-genome sequencing and comparative analysis. A full investigation of allelic variation in wild progenitor, traditional landrace and modern cultivars can be used to trace the selections in domestication and breeding. Increasing diversity from the landraces and wild sesame may benefit to the adaptation and further genetic improvement of modern cultivars.

## Methods

### Sampling and sequencing

All the samples were obtained from a large collection of ∼7,000 sesame accessions preserved at the China National Gene Bank, Oilcrops Research Institute, Chinese Academy of Agricultural Sciences. We selected 405 traditional landraces and 95 modern cultivars from China, as well as 205 accessions collected from 28 other countries. Detailed information including the geographical origin and sequencing coverage of the 705 accessions is listed in Supplementary Data 1. The 705 cultivated samples were maintained by self-pollination for at least four generations before sequencing and phenotyping. The genomic DNA was prepared from a single plant of each accession for sequencing and the library was constructed with an insert size of ∼300 bp. All the sesame varieties were sequenced on the Illumina HiSeq2500, which generated 96-bp paired-end reads. Two representative landraces, Baizhima and Mishuozhima, were deep sequenced with ∼70 coverage and *de novo* assembled.

### Read alignment and SNP calling

The paired-end reads were aligned against the sesame reference genome using the SMALT software ( http://www.sanger.ac.uk/resources/software/smalt/) with the parameter ‘−i 700 −j 50 −m 60'. Aligned reads were picked up with a cutoff of minimum 96% identity over 92% consecutive nucleotides in a read. Only the uniquely aligned reads that were mapped to unique locations in the reference genome were retained. These reads were used to call the single-base pair genotypes of the consensus sequences across the whole sesame genome by using the Ssaha Pileup package ( http://www.sanger.ac.uk/resources/software/ssaha2/). SNP identification and genotype calling were performed based on the outputs from the Ssaha Pileup package. The low-quality bases (base-quality *Q* score in Phred scale <25) were removed and those called sites with conflicting genotypes among different reads were also excluded. In addition, we required that the overall depth in each site was <180, to avoid mapping to regions with copy number variation. After that, the single-base pair genotypes of the 705 sesame accessions were integrated together for the SNP identification. Discrepancies with the sesame reference genome were called as candidate SNPs. Unreliable SNP sites were then filtered—the candidate SNP loci were required to be bi-allelic and all the singleton SNPs were excluded.

There were many missing genotypes generated after genotype calling from whole-genome resequencing data. The *k*-nearest neighbour-based method was used for missing data imputation (see http://www.ncgr.ac.cn/fimg/down.html). The imputation of the SNP genotypes of 705 sesame varieties reduced missing genotype calls from 47.9% to 3.7%. In total, 10 sesame accessions were randomly selected for additional whole-genome sequencing (∼16 × coverage for each). The sequence data of the 10 ten accessions was used to evaluate the identified SNPs. The overall concordance between them was estimated to be 97.8%. The specificity and missing data rate before and after imputation are presented in Supplementary Table 3.

### Sequence assembly and comparison

The genomes of two cultivated sesame varieties were assembled by using the SOAPdenovo2 package (version 2.04) and Gapcloser (version 1.12)^43^. The N50 length of the final assembly was calculated with all small contigs of <200 bp excluded. The contig sequences of the whole-genome assemblies were anchored to the sesame reference genome sequence using the software MUMmer^44^ and the sequence variants were further called using the diffseq programme in the EMBOSS package^45^. The potential effects of the sequence variants were predicted based on the genome annotation of the sesame reference genome from GFF files. The software FGeneSH was used for the gene structure prediction in the two genomes^46^. The *de novo* assemblies, the BLAST searches and genome-wide analysis of all the coding variants are available at the Sesame Haplotype Map Project database ( http://www.ncgr.ac.cn/SesameHapMap).

### Planting and phenotyping

For the seed sowing, normal and well-rounded seeds of each accession (∼3 g) were selected manually for the field experiments. The field was deeply ploughed and tilled to ensure that the soil conditions and other field management procedures were equal for all the accessions evaluated in this trial. The proper soil moisture content (15%–20%) was achieved through field irrigation ∼1–2 weeks before sowing the field. The row spacing was measured before sowing and land rolling were conducted and finished in the same day. Zhongzhi-13, one of the most widely grown varieties in China, was planted as a control. For the seedling thinning, seedlings at the four-leaf stage were manually thinned out to achieve an equal density of 120,000 individuals per hectare (40 cm in raw, 20 cm in row). For phenotyping, the collection of 705 cultivated accessions was planted in four environmental conditions: (i) from May to September 2013 in Wuhan, Hubei Province, China, at N 30.57°, E 114.30°, altitude 27 m; (ii) from August to November 2013 in Nanning, Guangxi Province, China, at N 23.17°, E 107.55°, altitude 220 m; (iii) from November 2013 to February 2014 in Sanya, Hainan Province, China, at N 18.23°, E 109.50°, altitude 7 m; and (iv) from May to September 2014 in Luohe, Henan Province, China, at N 33.40°, E 113.33°, altitude 76 m. Five individuals from each accession were randomly labelled and screened for all the trait measurements. In all four environments, the phenotyping procedure and scoring standard were the same. A total of 169 sets of phenotypes in all four environments including the yield formation, growth period, plant and capsule variation, and disease resistance ability were systematically characterized and scored. The early flowering date of each accession was recorded daily as the number of days from sowing to the observation of the first flower on 50% of the individuals. Flower and leaf-related traits were observed and measured in the full-bloom stage. Yield-related traits were measured manually in the laboratory after harvest. The seed inside of the capsules were carefully poured out and counted. Seed composition was detected after all the yield-related traits were recorded.

### Measurement of the seed composition

Approximately 15 g mature and well-rounded seeds were chosen for each accession. After drying at 80 °C for 2 h, we milled the seeds to a fine powder with an electric grinder and the solid fractions were excluded through a 0.25-mm sieve. All the powders were divided into two sub-samples and were measured at the same time. Protein content was determined by the standard Kjeldahl procedure using a Kjeltec 8400 Analyzer (Foss, Sweden) according to the user manual. For the oil content measurement, 1 g seed powder was weighted and soaked in mineral ether for 12 h and then it was dried at 105 °C for 3 h and weighted again. The oil content was calculated by subtracting the weight of the seed powder. All oil components in seed are extracted by mineral ether and oil content in the seed is calculated by the change of the mass. Therefore, the oil content in this work was the absolute content. To detect the content of sesamin and sesamolin, 200 mg powder was weighted and dissolved in 80% ethanol. Sesamin and sesamolin in the extracted solutions were quantified by Agilent 1100 HPLC^47^. The oil components in sesame seeds were extracted by mineral ether and were further esterified by KOH-methanol solution (0.4 M). After adding the distilled water, the samples were centrifuged. The fatty acid composition were measured with a 7890A gas chromatogram (Agilent Technologies)^48^. The content of each fatty acid is expressed as its percentage (%) among total fatty acids. Approximately 1 g seed coat was stripped from whole seeds manually and then milled to fine powder. The lignin content in the seed coat was estimated from the standard curve using ultraviolet spectrophotometer^49^.

### Population genetics analysis and GWAS study

Simple matching coefficients were used to construct phylogenetic trees using the software PHYLIP^50^. Principal component analysis of the population was performed using the software EIGENSOFT^51^. The sequence diversity statistics (*π*) were computed in each 100-kb window of the sesame genome. Association analysis was performed using the EMMAX software package^52^ and the matrix of pair-wise genetic distance derived from simple matching coefficients was used as the variance–covariance matrix of the random effect.

Permutation tests were used to help define the threshold^53^. We randomly selected ten traits, reshuffled the original phenotype data and then performed association analysis using EMMAX with the same parameters. There ought to be no real associations between the SNPs and the ‘simulated' phenotypes; therefore, all the SNPs passing the threshold should be false positives. A total of 100 permutation analyses were performed, which detected 15 ‘association signals' passing the whole-genome significance cutoff of 10^−6^ and 1 ‘association signal' passing the cutoff of 10^−7^. GWAS study on 169 real phenotypes identified a total of 549 association signals passing the threshold of 10^−6^ and 303 signals passing the threshold of 10^−7^, which suggested an FDR <0.05 for the threshold of 10^−6^ and an FDR <0.01 for the threshold of 10^−7^. The significance threshold was determined using a modified Bonferroni correction (Genetic type 1 Error Calculator, version 0.2)^54^. The effective number of independent SNPs were estimated to be 469,175 and the threshold was estimated to be approximately *P*=10^−7^.

Multiple linear regressions were performed to examine the effects of multiple alleles in multiple loci underlying the complex traits including the oil content, plant height and flowering time, using the proc reg procedure in SAS. Before fitting the model, each marker was recoded (the value 0 was used for the reference alleles qand the value 1 was used for the alternative alleles). The value *R*^2^ was calculated as the proportion of the total phenotypic variation explained by the regression model.

### Expression pattern of *SiPPO* and *SiNST1*

Leaf, root and stem were collected from two accessions (G404, ‘C' allele and G620, ‘A' allele) in vegetables stage. Flower was collected in the beginning of flowering. The capsules were marked by threads of different colours from the first day of flowering. Developing seeds were carefully collected manually from the capsules after the ovules were pollinated at 5, 8, 11, 14, 17 and 20 days. The total RNAs of the fresh samples were extracted using an EASYspin Plus kit (Aidlab) according to the manufacturer's instructions. The RNA was treated with DNaseI and reverse transcribed with oligo (dT23) primer using the HiScript II 1st Strand cDNA Synthesis kit (Vazyme). The qRT–PCR experiments were performed with gene-specific primers and probes in the reaction system of Premix Ex Taq (Takara) on the CFX384 Real-Time System (Bio-Rad) according to the manufacturer's instructions. The qRT–PCR assay was performed in triplicate and the sesame actin7 gene (SIN_1006268) was used as an internal control. The primers used for gene amplification included *SiPPO*-F: 5′-GGAGTAAAGAAGAGAAAGAAG-3′, *SiPPO*-R: 5′-GGGTTTACTGCAATCATAC-3′; *SiNST1*-F: 5′-GCAACAGAGATTGTCATC-3′, *SiNST1*-R: 5′-GCTCCAAAGATCACATTC-3′; and *SiActin7*-F: 5′-CTGTCAACAGAATTGGGTG-3′, *SiActin7*-R: 5′-GCAACTGGGATGATATGG-3′. The probes used in the qRT–PCR included the *SiPPO*-probe: FAM-5′-TTCGTCTACCAGCAACACCTCTT-3′-BHQ1, the *SiNST1*-probe: FAM-5′-CCTACGGTACGGCTACTCACG-3′-BHQ1 and the *SiActin7*-probe: FAM-5′-CCTTCTACAATGAACTTCGTGTGGC-3′-BHQ1. The reads per kilobase of transcript per million mapped read values, which represented the expression levels of *SiPPO* in white (G610, ‘A' allele) and black (G122, ‘C' allele) sesame seeds 10, 20, 25 and 30 days after pollination were calculated from the RNA-seq data (NCBI ID: SRA122023)^7^.

## Additional information

**Accession codes.** The DNA sequencing data are deposited in the European Nucleotide Archive (http://www.ebi.ac.uk/ena/data/view) under accession numbers PRJEB8078. The genotypic data set of the 705 sesame varieties for GWAS and the two genome assemblies are available at the Sesame Haplotype Map Project database (http://www.ncgr.ac.cn/SesameHapMap).

**How to cite this article:** Wei, X. *et al.* Genetic discovery for oil production and quality in sesame. *Nat. Commun.* 6:8609 doi: 10.1038/ncomms9609 (2015).

## Supplementary Material

Supplementary Figures and Tables Supplementary Figures 1-20 and Supplementary Tables 1-5

Supplementary Data Set 1 The list of 705 accessions sampled in the collection

Supplementary Data Set 2 The summary of quantitative traits phenotyped in four environments

Supplementary Data Set 3 The full lists of all significant associations

Supplementary Data Set 4 The candidate causative genes in the associated loci

Supplementary Data Set 5 Oil related genes in sesame and their orthologs in Arabidopsis and maize

Supplementary Data Set 6 The loci selected in modern breeding
