# Supplementary material for: Genetic discovery for oil production and quality in sesame
Source: Nat Commun. 2015 Oct 19;6:8609. doi: 10.1038/ncomms9609 (PMC4634326; doi:10.1038/ncomms9609)
Supplement: Supplementary Figures and Tables — Supplementary Figures 1-20 and Supplementary Tables 1-5 [file ncomms9609-s1.pdf]

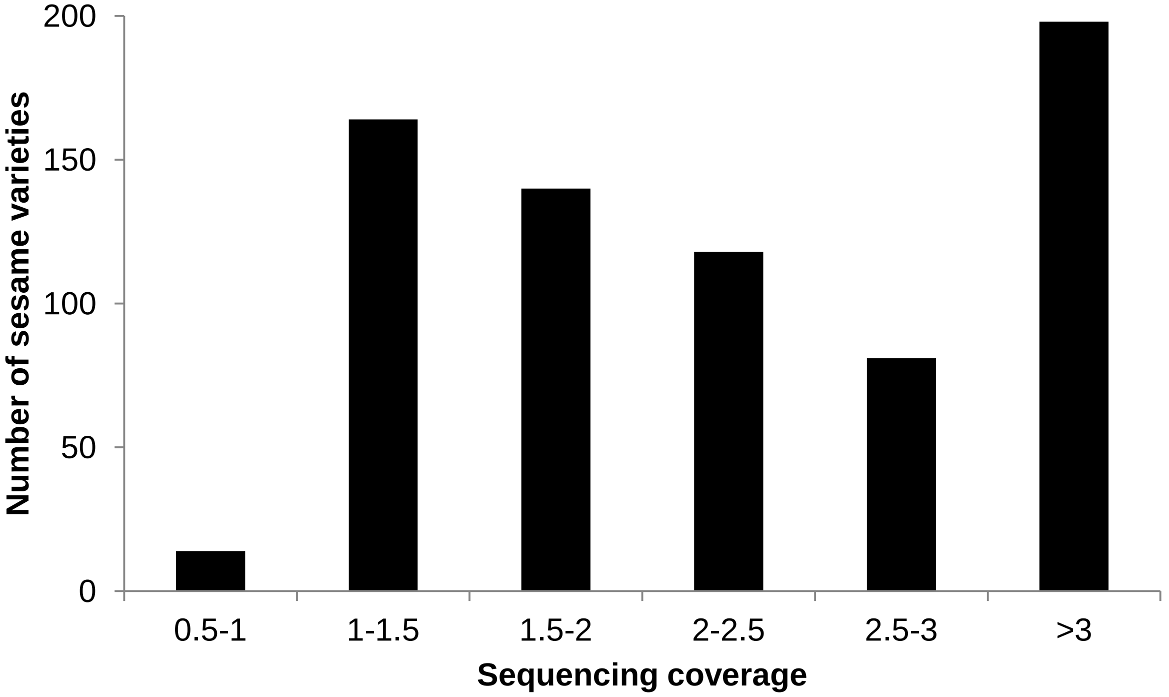

Supplementary Figure 1 Sequencing coverage of 705 sesame accessions. The genomes of the sesame varieties were sequenced on the Illumina HiSeq2500 platform. An average of 2.6 fold genome coverage of 2x96bp Illumina reads was generated for each sample.

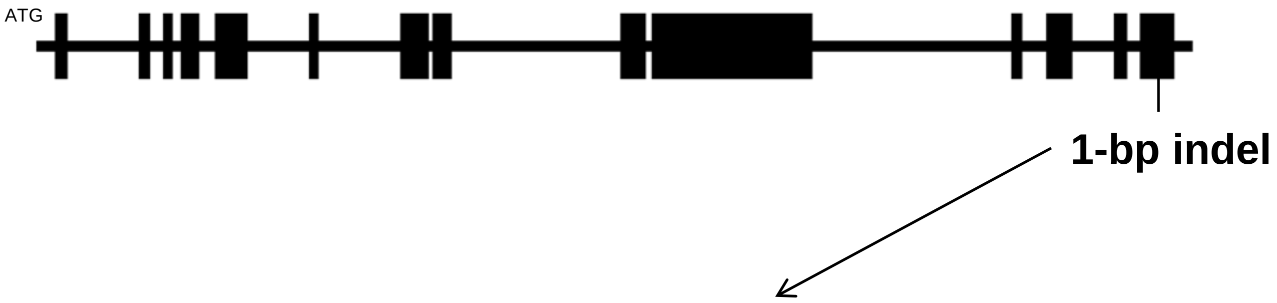

Reference genome : ATG......CTGCAGCAACGGAATTAGGCTGTACTATATCCATTTGA
Mishuozhima: ATG......CTGCAGCAACGGAATTAGGCTGTACTATATCCATTTGA
Baizhima: ATG......CTGCAGCAACGGAAT-AGGCTGTACTATATCCATTTGA

Supplementary Figure 2 Large-effect variants detected in the flowering gene *SiGI* from three whole-genome assemblies.

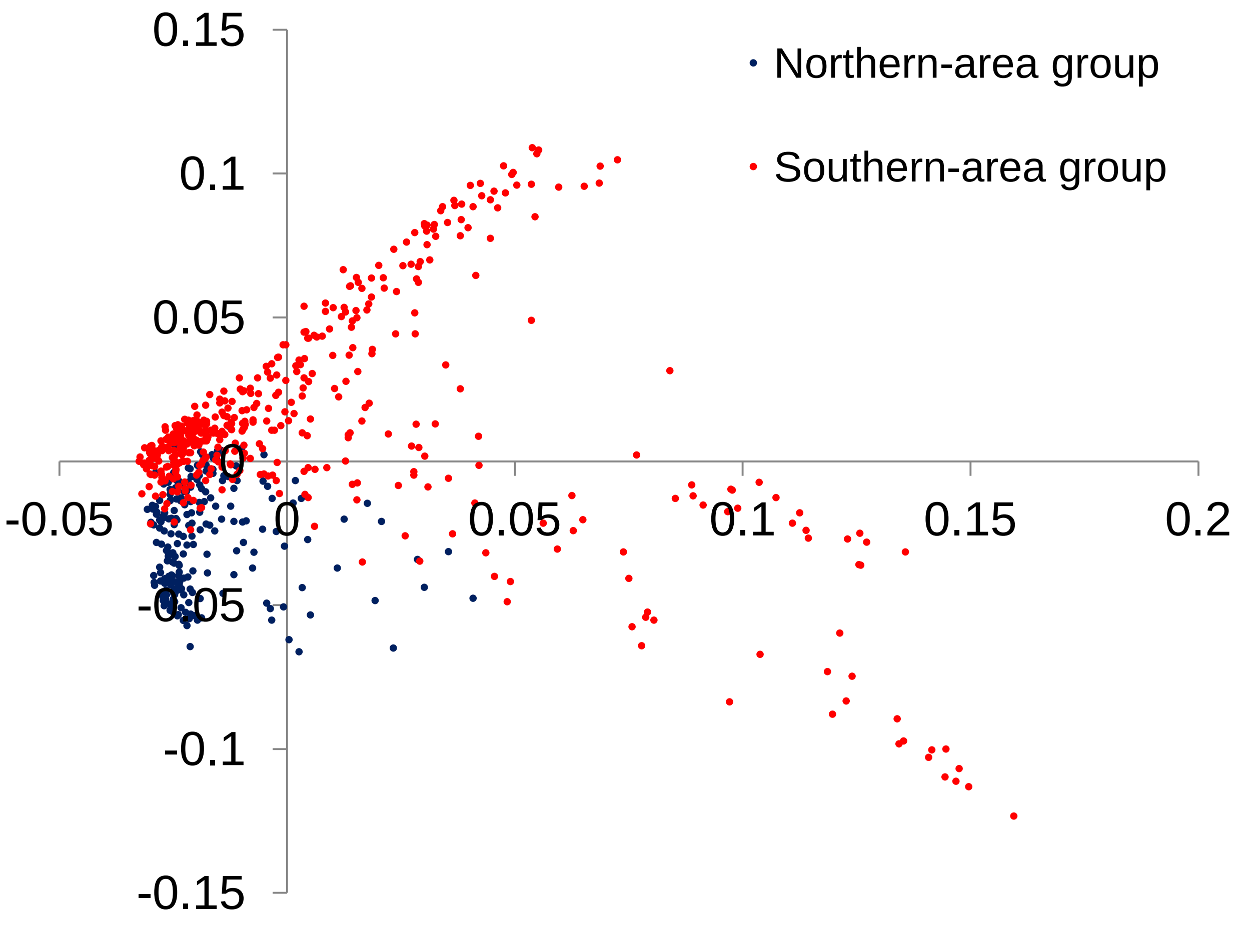

Supplementary Figure 3 Principal-component analysis of 705 sesame varieties. The two recognizable groups are colored in red and blue, respectively. The X-axis represents the first principal component, and the Y-axis shows the second principal component.

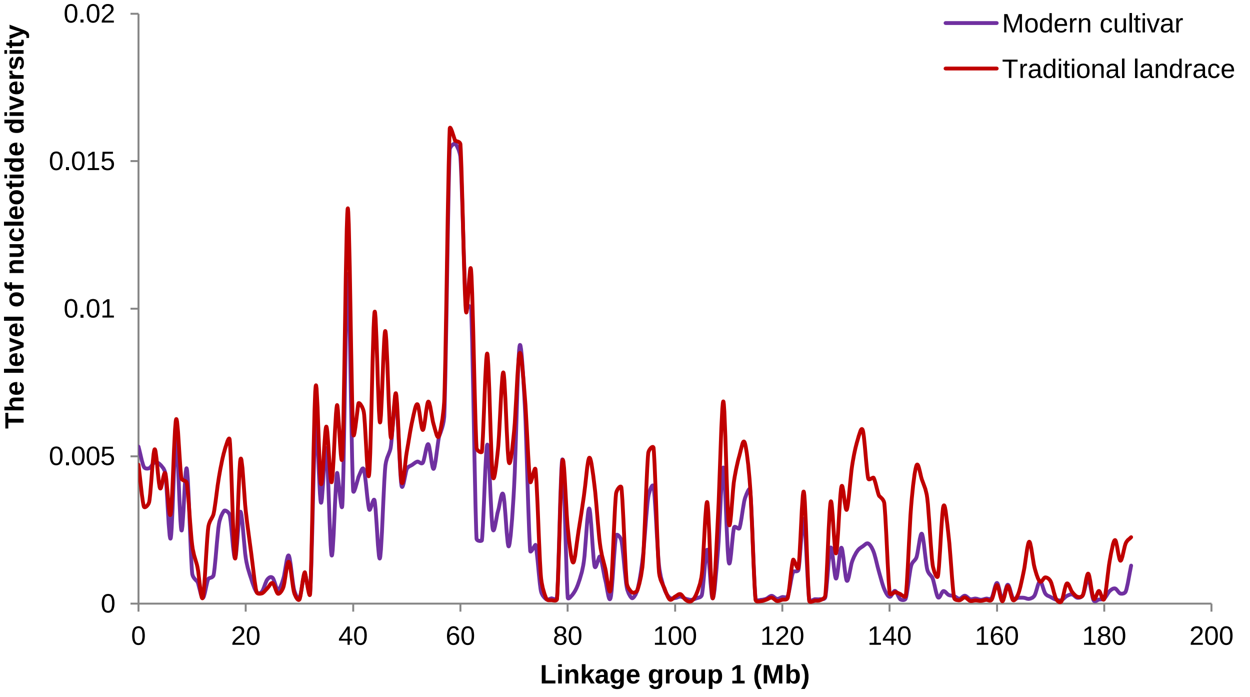

Supplementary Figure 4 The level of nucleotide diversity of modern cultivars and traditional landraces in linkage group 1.

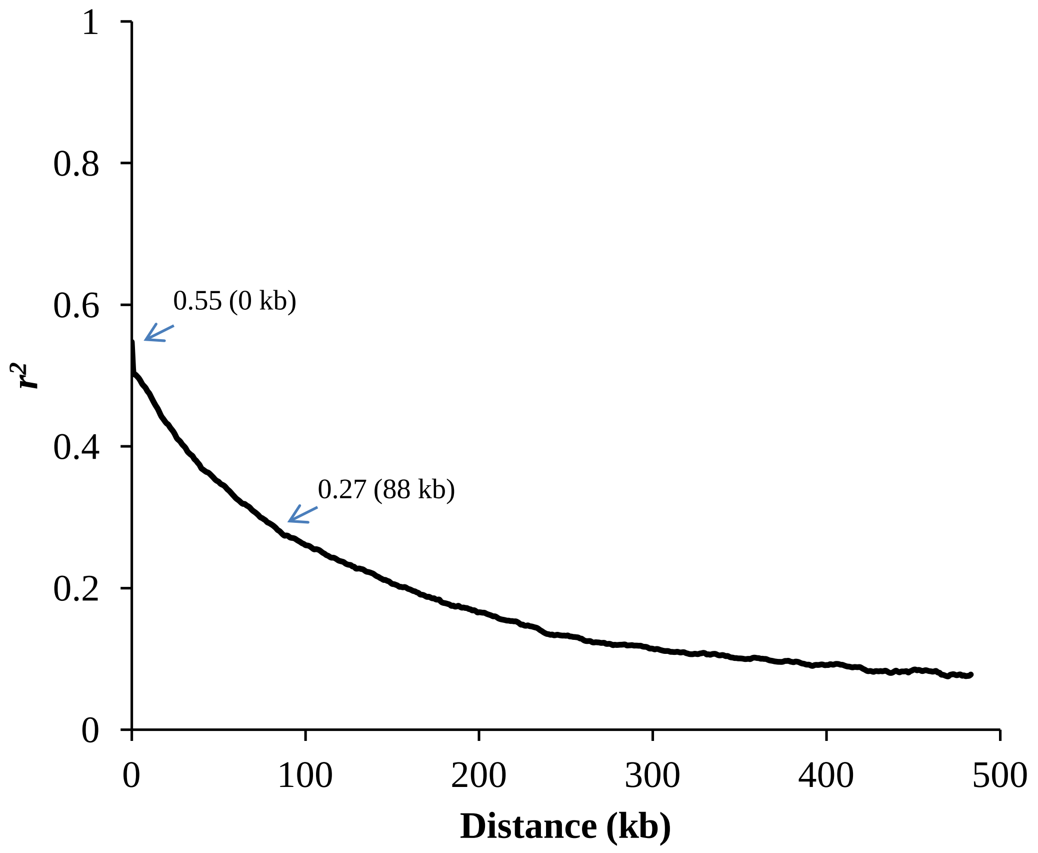

Supplementary Figure 5 Genome-wide average LD decay estimated from 705 sesame accessions.

a

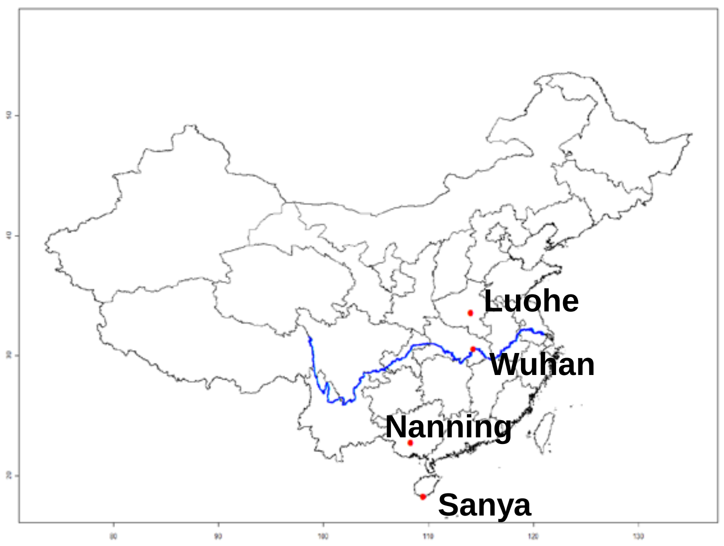

b

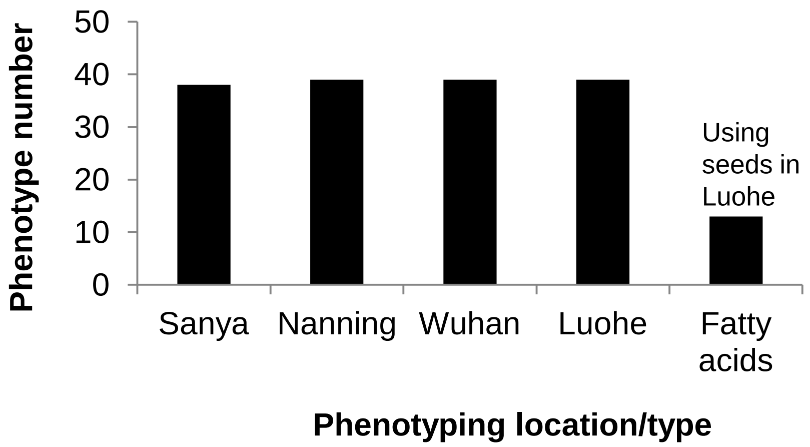

c

Phenotype Number	Sanya	Luohe	Nanning	Wuhan
35	+	+	+	+
2	+	-	+	+
2	-	+	+	+
1	+	-	-	-
16	-	+	-	-

Supplementary Figure 6 The 705 sesame accessions were planted in four agro-ecologically diverse locations in China for extensive phenotyping. (**a**) The geographic locations of four phenotyping sites. (**b**) The number of traits that were phenotyped in this study. (**c**) The number of the phenotypes that were investigated in each site.

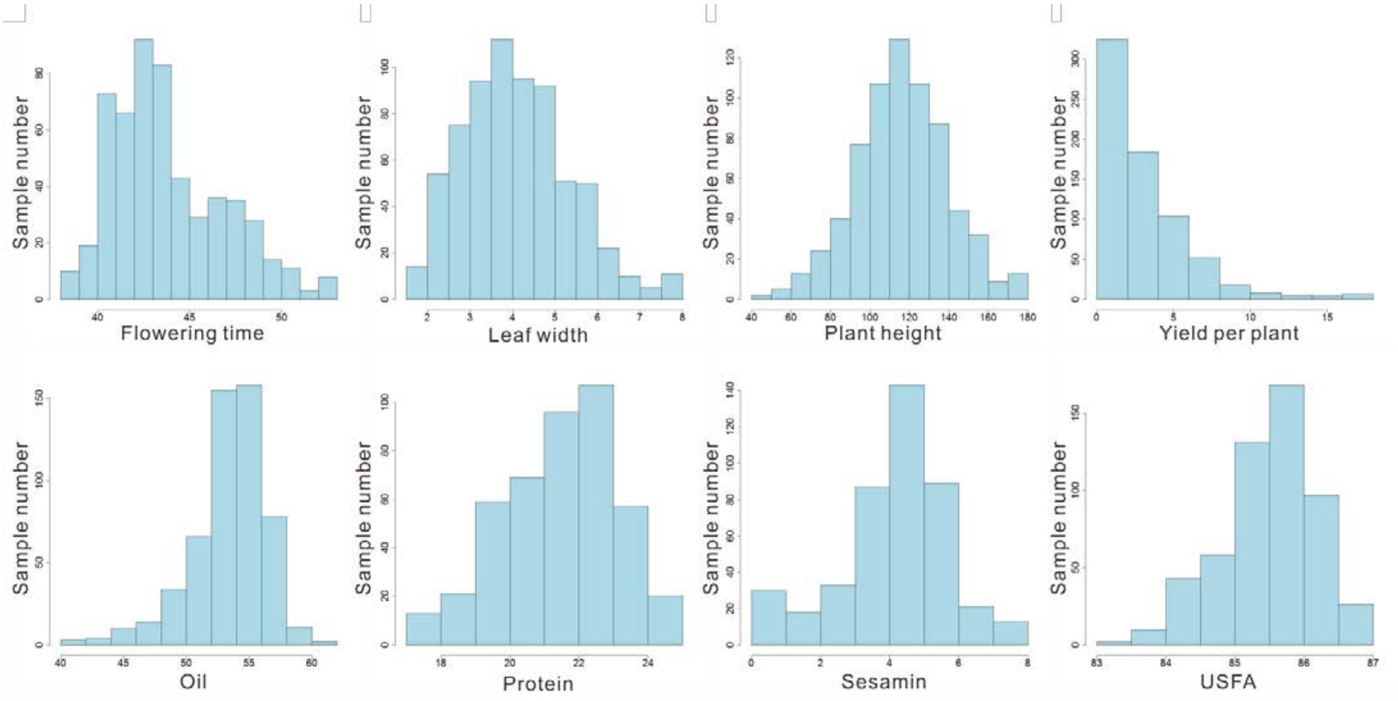

Supplementary Figure 7 Frequency distribution of phenotypic variation of eight agronomic traits in 705 sesame varieties.

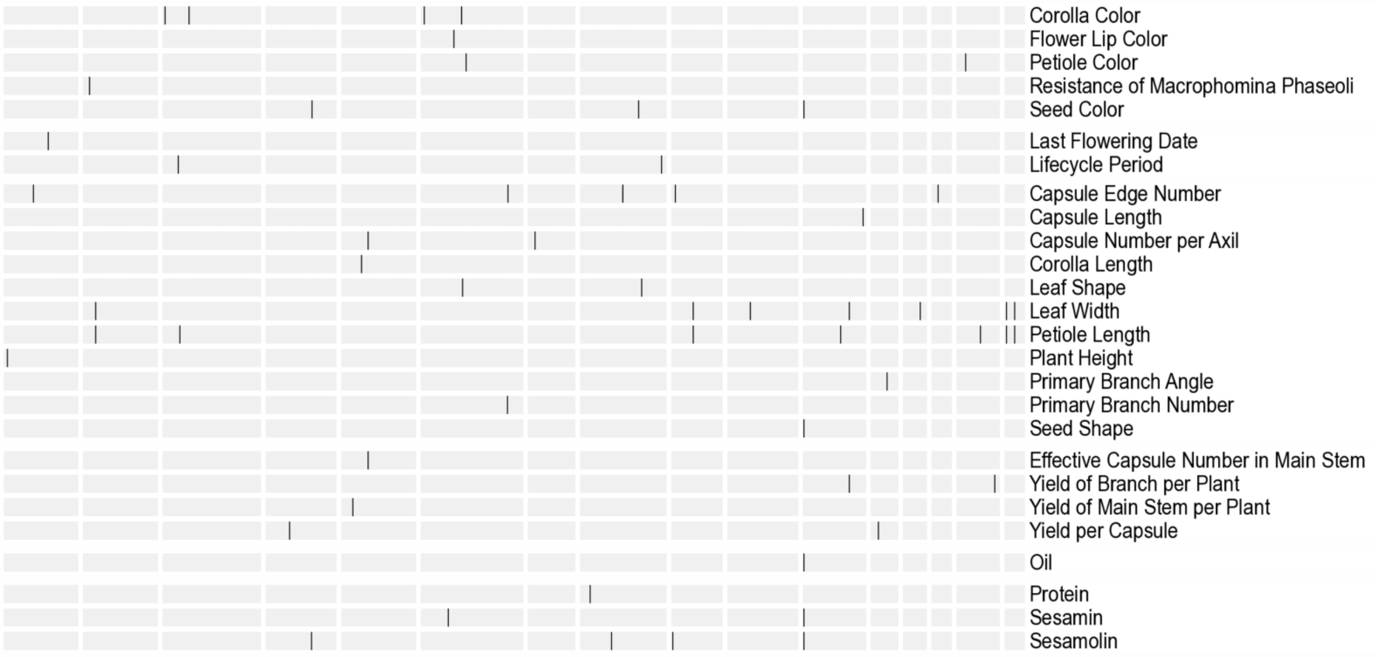

Supplementary Figure 8 Large-scale genetic discovery of agronomic traits phenotyped in Nanning. For each trait, the associated loci ($P<1\times10^{-6}$) are indicated in the genome.

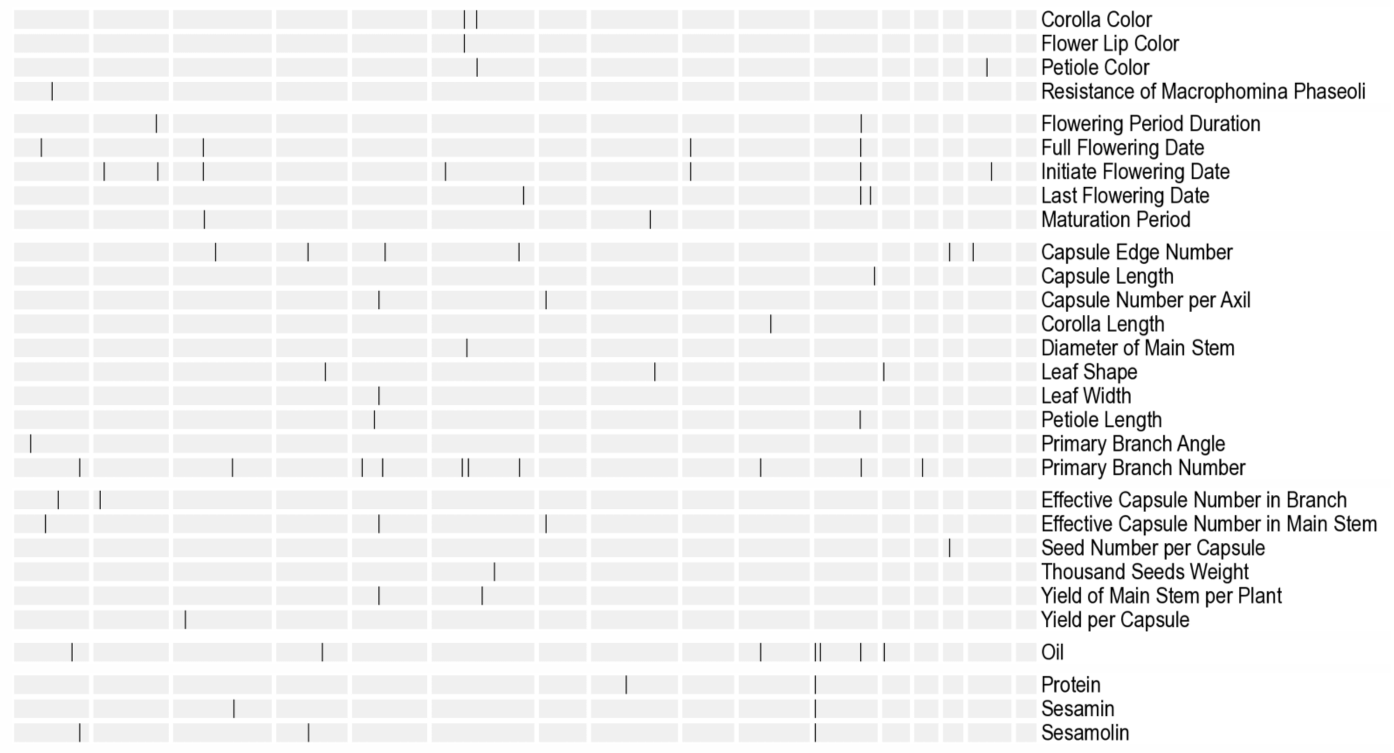

Supplementary Figure 9 Large-scale genetic discovery of agronomic traits phenotyped in Luohe. For each trait, the associated loci ($P<1\times10^{-6}$) are indicated in the genome.

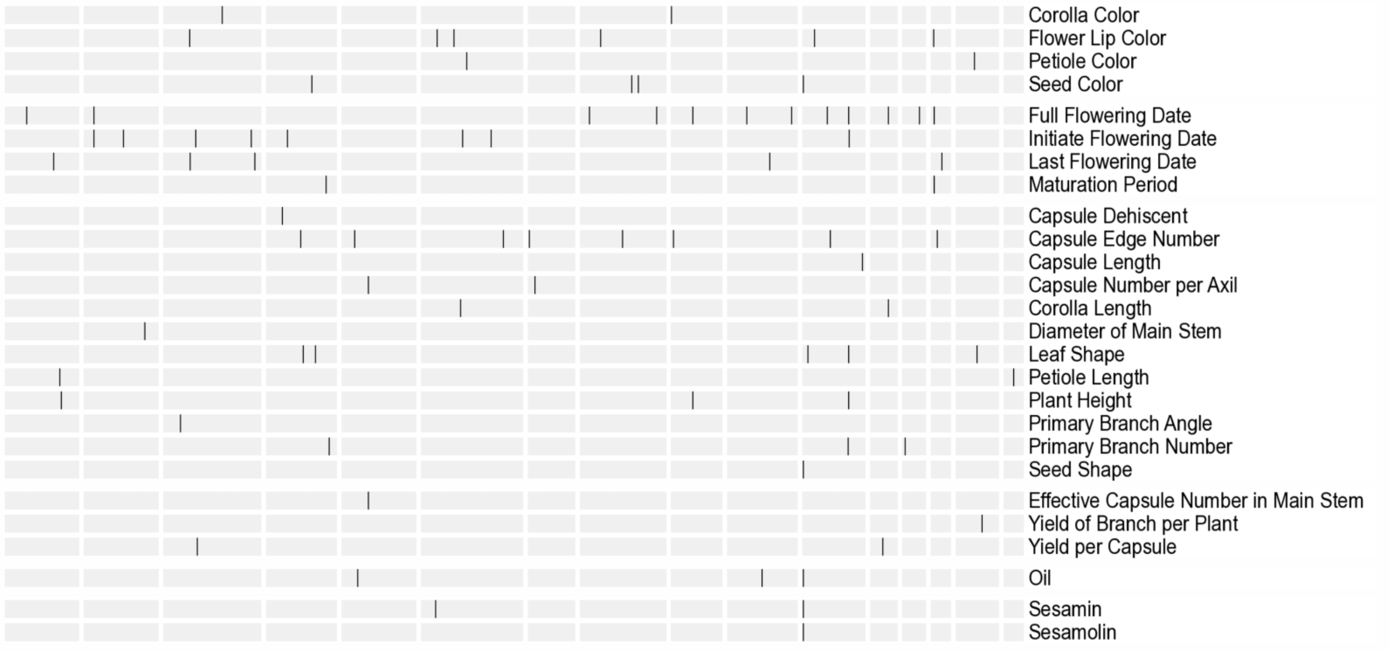

Supplementary Figure 10 Large-scale genetic discovery of agronomic traits phenotyped in Wuhan. For each trait, the associated loci ($P<1\times10^{-6}$) are indicated in the genome.

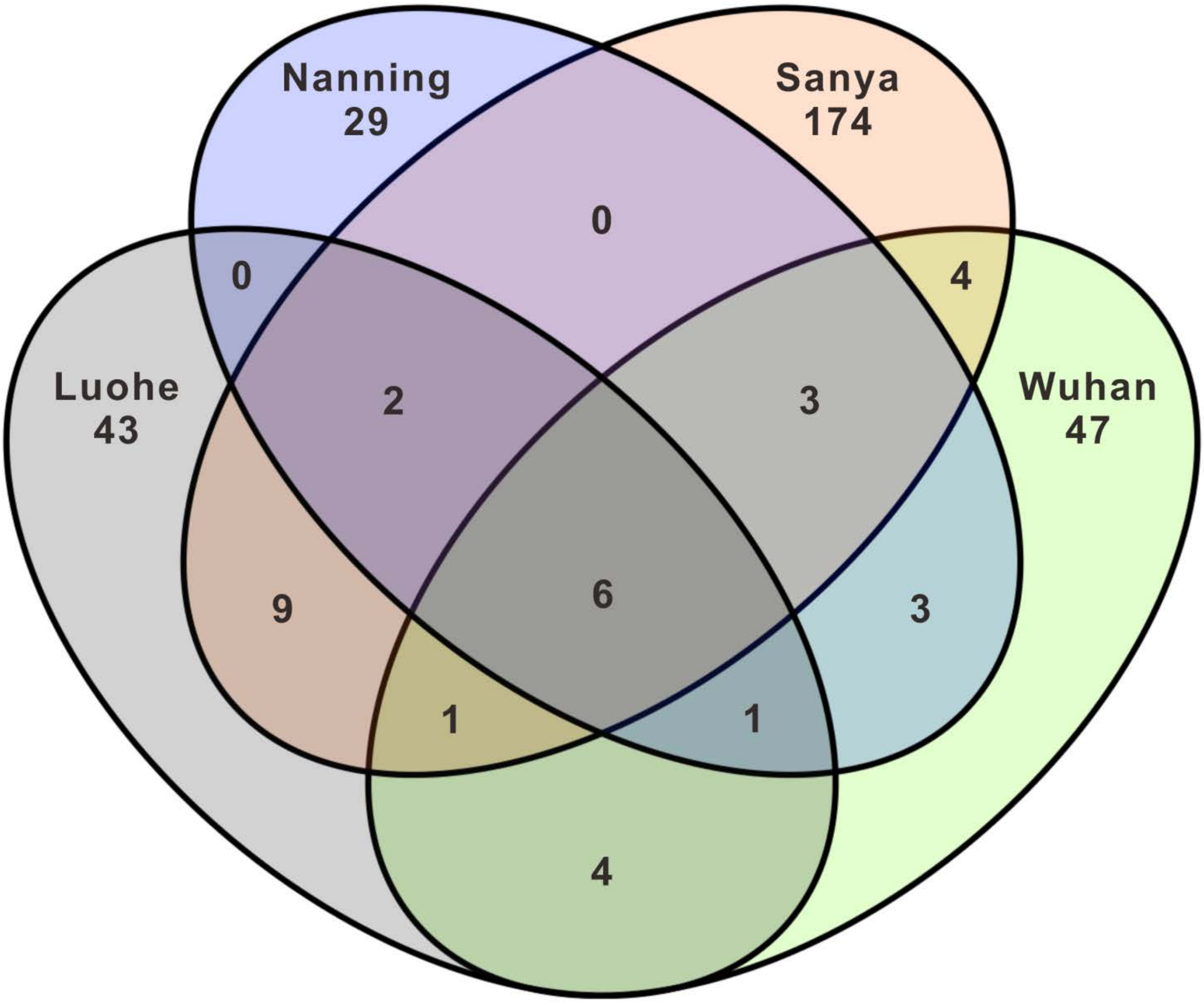

Supplementary Figure 11 The Venn diagram of GWAS loci underlying the traits phenotyped in four environments.

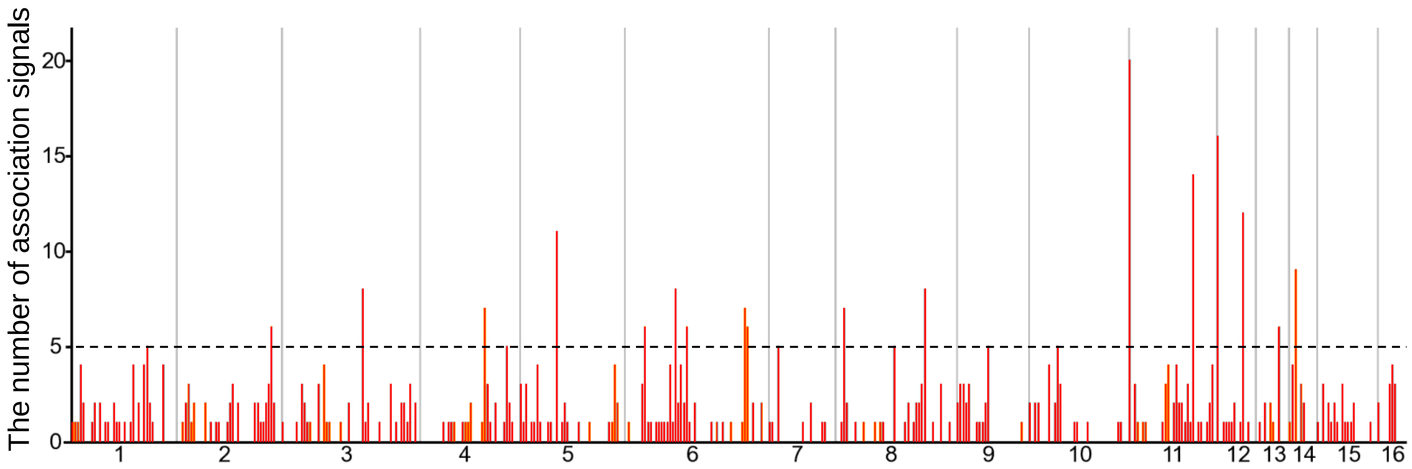

Supplementary Figure 12 The genome-wide distribution of the association signals in sesame, per 500 kb genome segment as a window. There are totally 17 hot spots with significantly more association signals than expected (> 5 associations per window, *P*<0.01, binomial test).

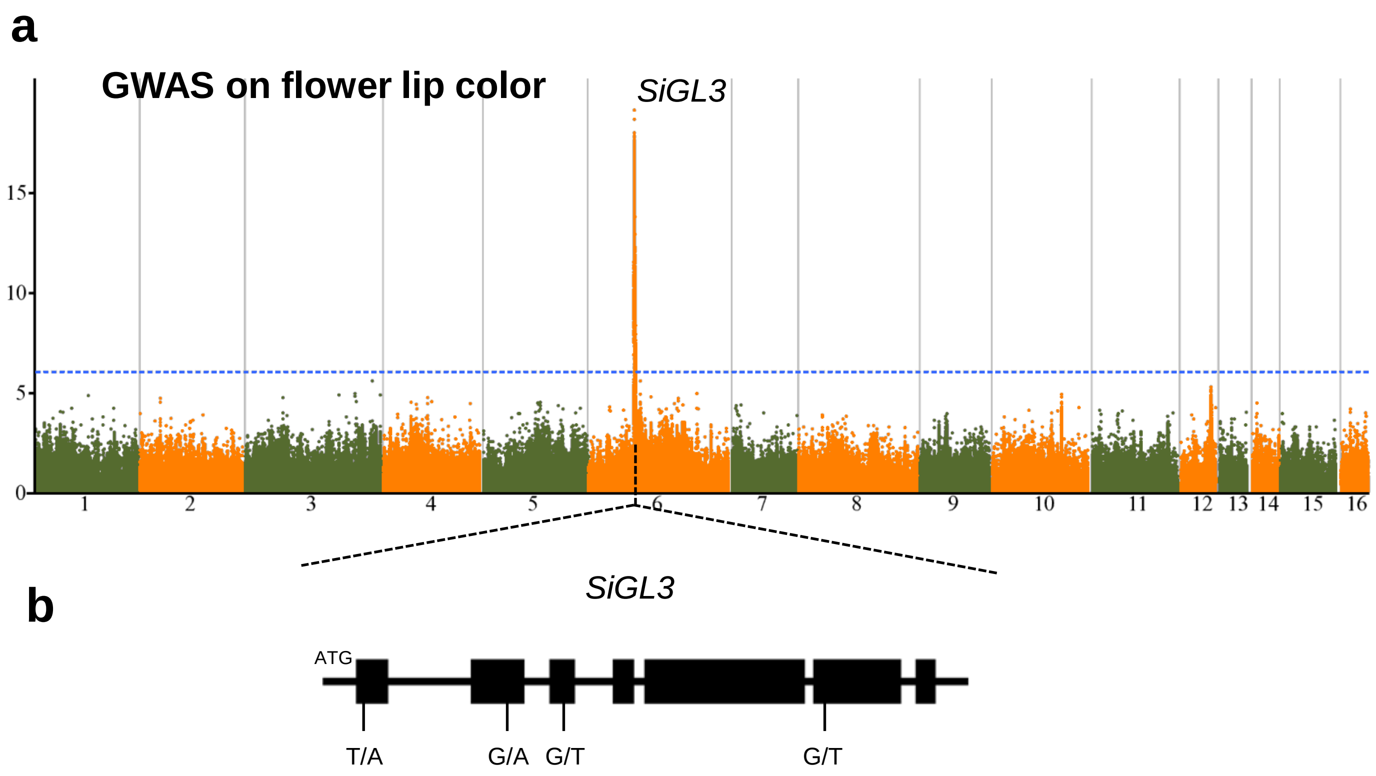

Supplementary Figure 13 Candidate causative genes and variants underlying flower lip color. (**a**) Negative $\log_{10}$ *P*-values for association of flower lip color phenotyped in Luohe (Y-axis) are plotted against SNP positions (X-axis). The genome-wide significant *P* value threshold (10^{-6}) is indicated by a horizontal dash-dot line. The candidate gene is indicated near the association peaks. (**b**) Candidate causative variants in *SiGL3*.

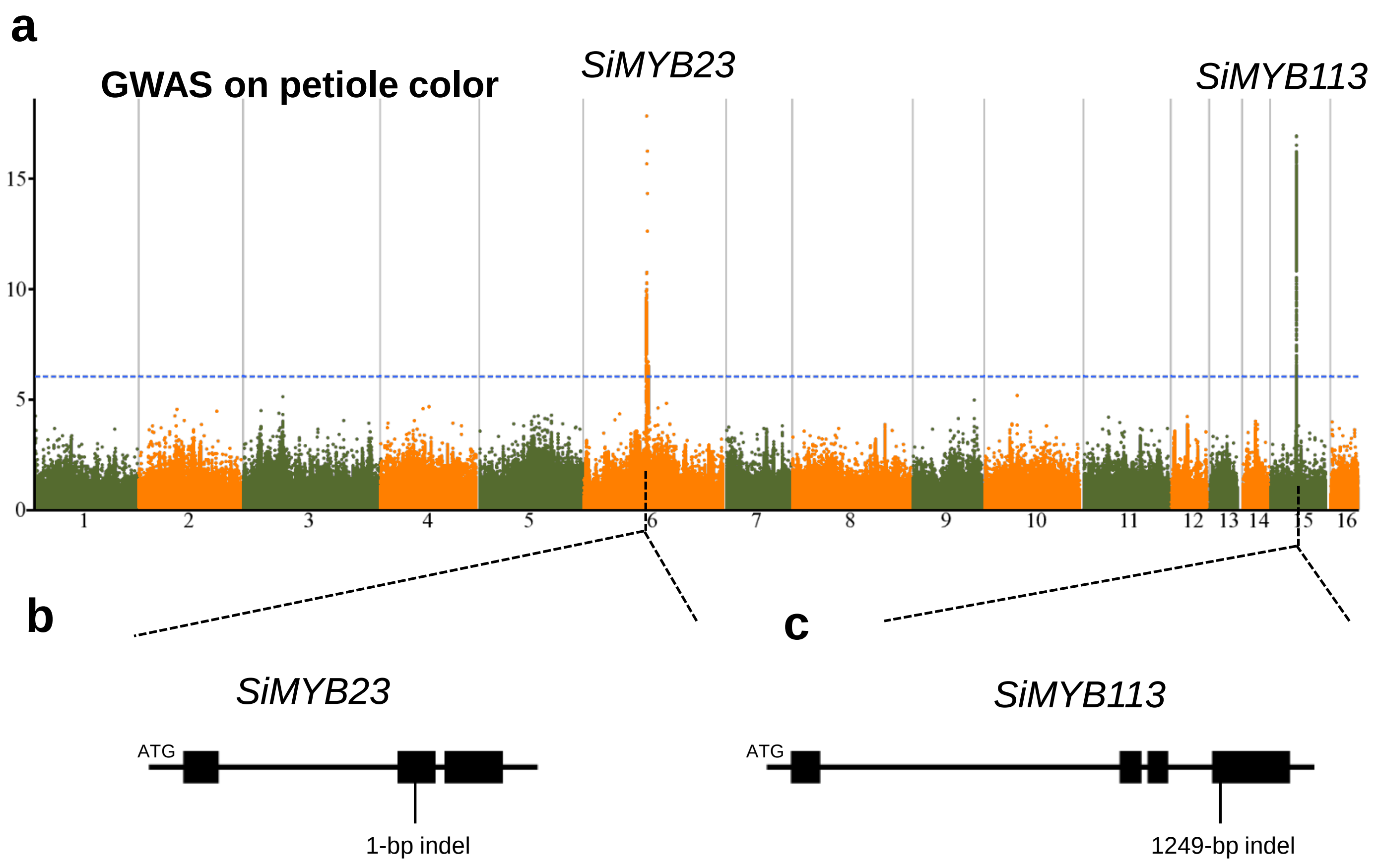

Supplementary Figure 14 Candidate causative genes and variants underlying petiole color. (**a**) Negative $\log_{10}$ *P*-values for association of petiole color phenotyped in Sanya (Y-axis) are plotted against SNP positions (X-axis). The genome-wide significant *P* value threshold (10^{-6}) is indicated by a horizontal dash-dot line. The candidate genes are indicated near the association peaks. (**b**) Candidate causative variant in *SiMYB23*. (**c**) Candidate causative variant in *SiMYB113*.

a

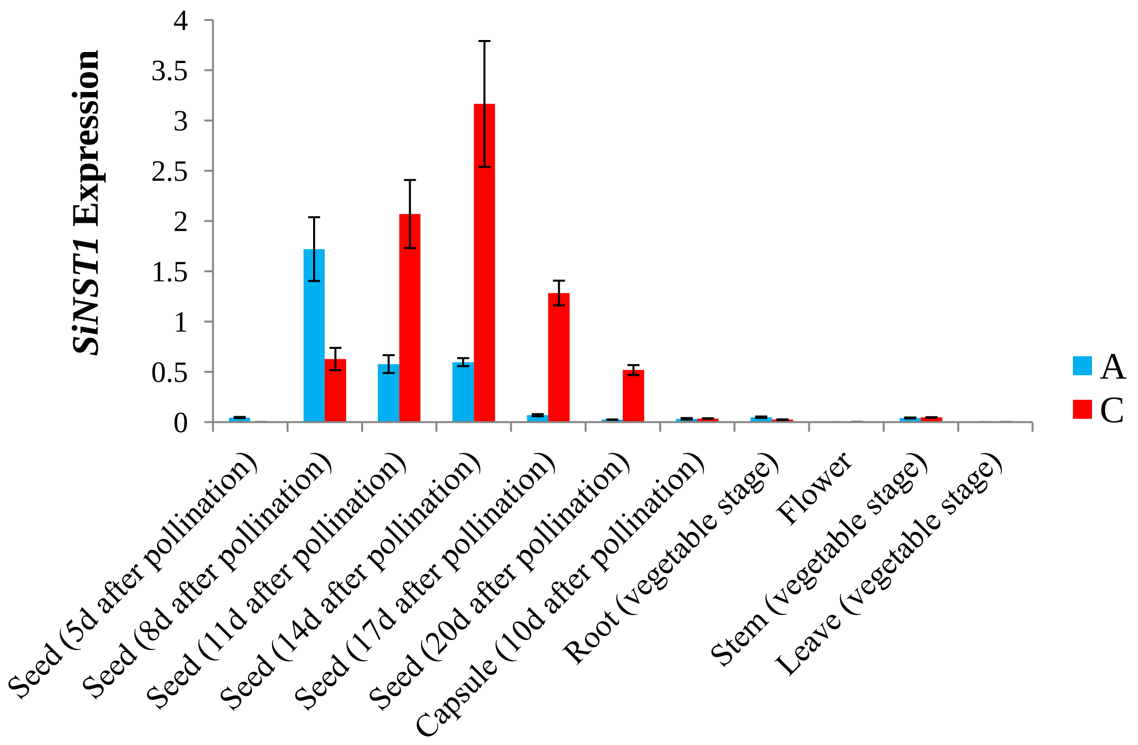

b

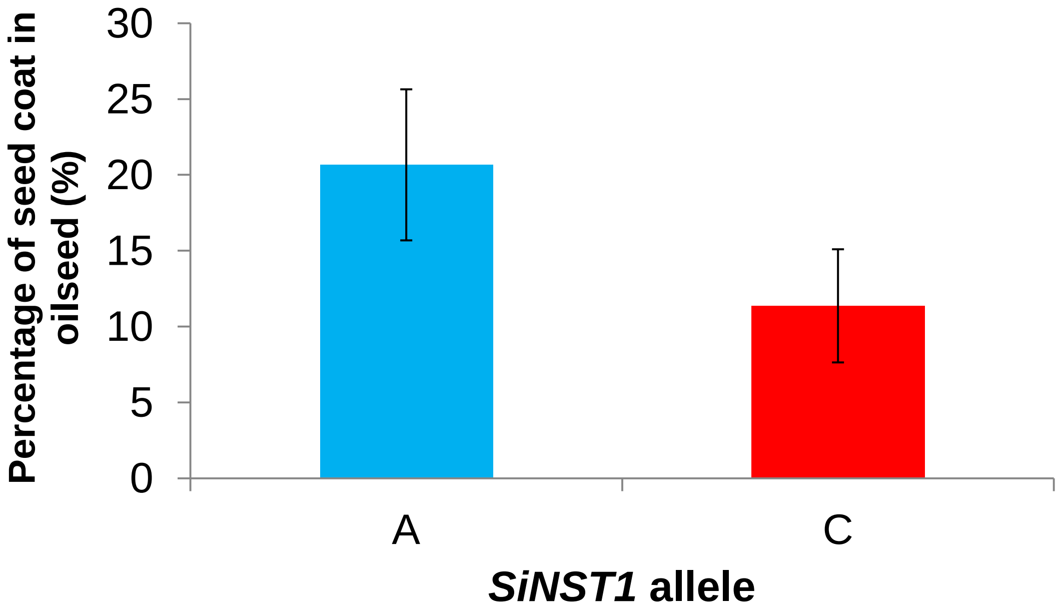

Supplementary Figure 15 A major gene *SiNST1* for both oil content and seed coat thickness in sesame. (**a**) The qPCR result of *SiNST1* in sesame. (**b**) The percentage of seed coat in sesame oilseeds. The varieties with “A” allele show much thicker seed coat than those with “C” allele significantly (*P*< 0.01). The bar indicates standard deviation.

a

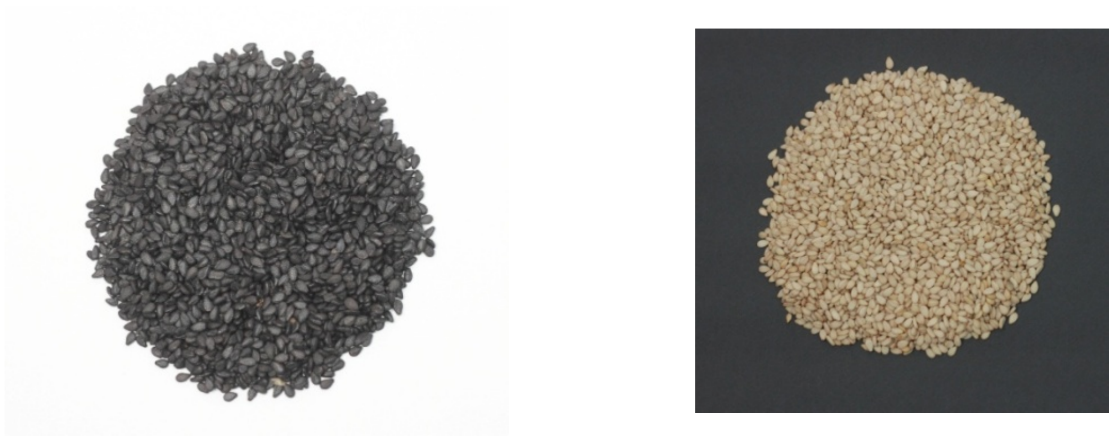

b **GWAS on seed coat color**

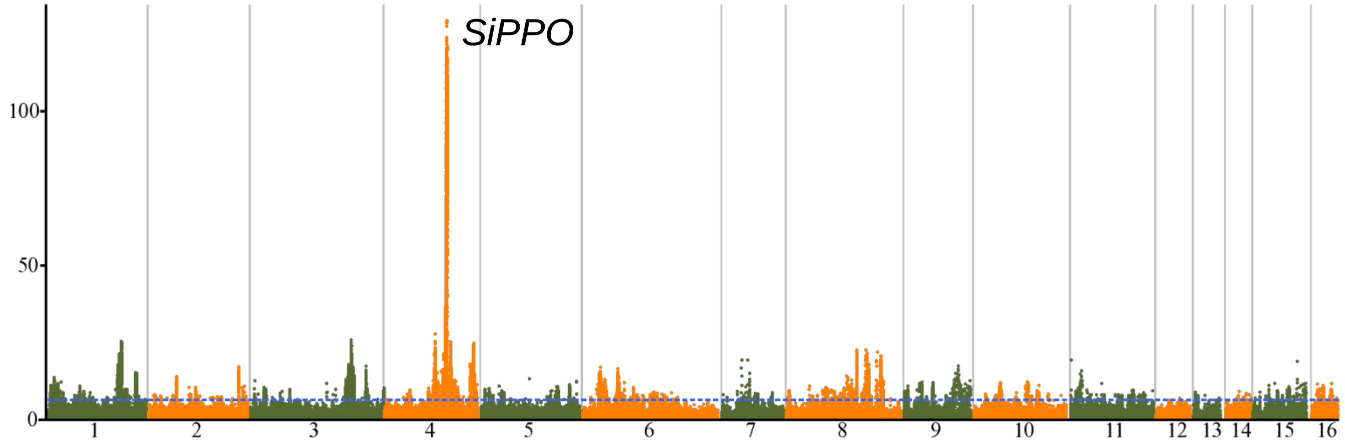

c

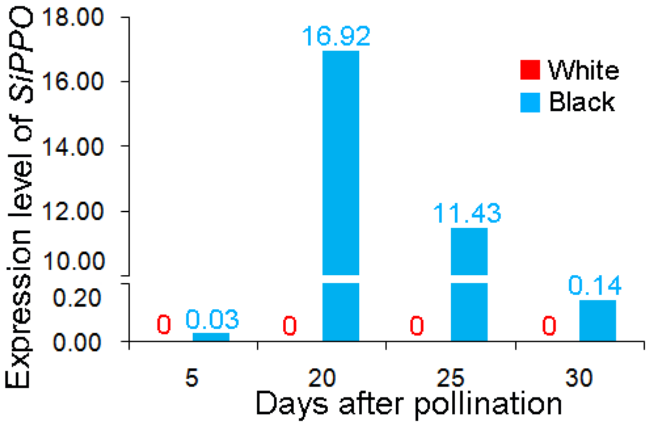

Supplementary Figure 16 A major gene *SiPPO* for both oil content and seed coat color in sesame. (**a**) Photo of the phenotypic change from black seed coat in wild sesame to white seed coat in modern cultivars. (**b**) Negative $\log_{10}$ *P*-values for association of seed coat color phenotyped in Sanya (Y-axis) are plotted against SNP positions (X-axis). The candidate gene is indicated near the association peaks. (**c**) Expression profiles of *SiPPO* in seeds. RPKM value of all sample is given above each bar. The white sesame accession (mutated allele) has no transcripts detected.

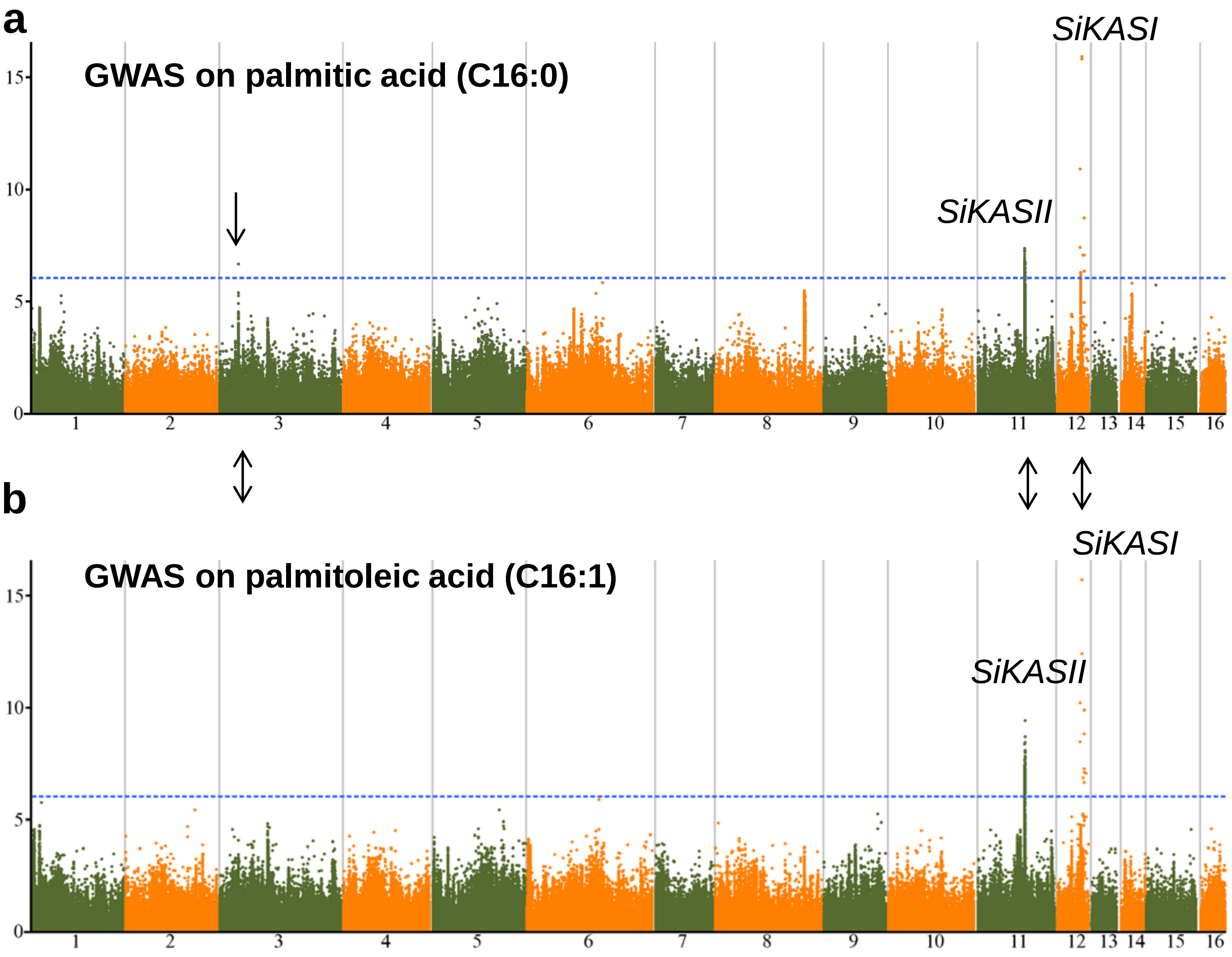

Supplementary Figure 17 Comparison of GWAS on two highly correlated traits palmitic acid (C16:0) composition and palmitoleic acid (C16:1) composition. (**a**) Negative $\log_{10}$ *P*-values for association of palmitic acid (C16:0) composition (Y-axis) are plotted against SNP positions (X-axis). The candidate genes are indicated. (**b**) Negative $\log_{10}$ *P*-values for association of palmitoleic acid (C16:1) composition (Y-axis) are plotted against SNP positions (X-axis). The candidate genes are indicated near the association peaks. The locus at the linkage group 3 for palmitic acid shows no association signal in GWAS for palmitoleic acid .

a

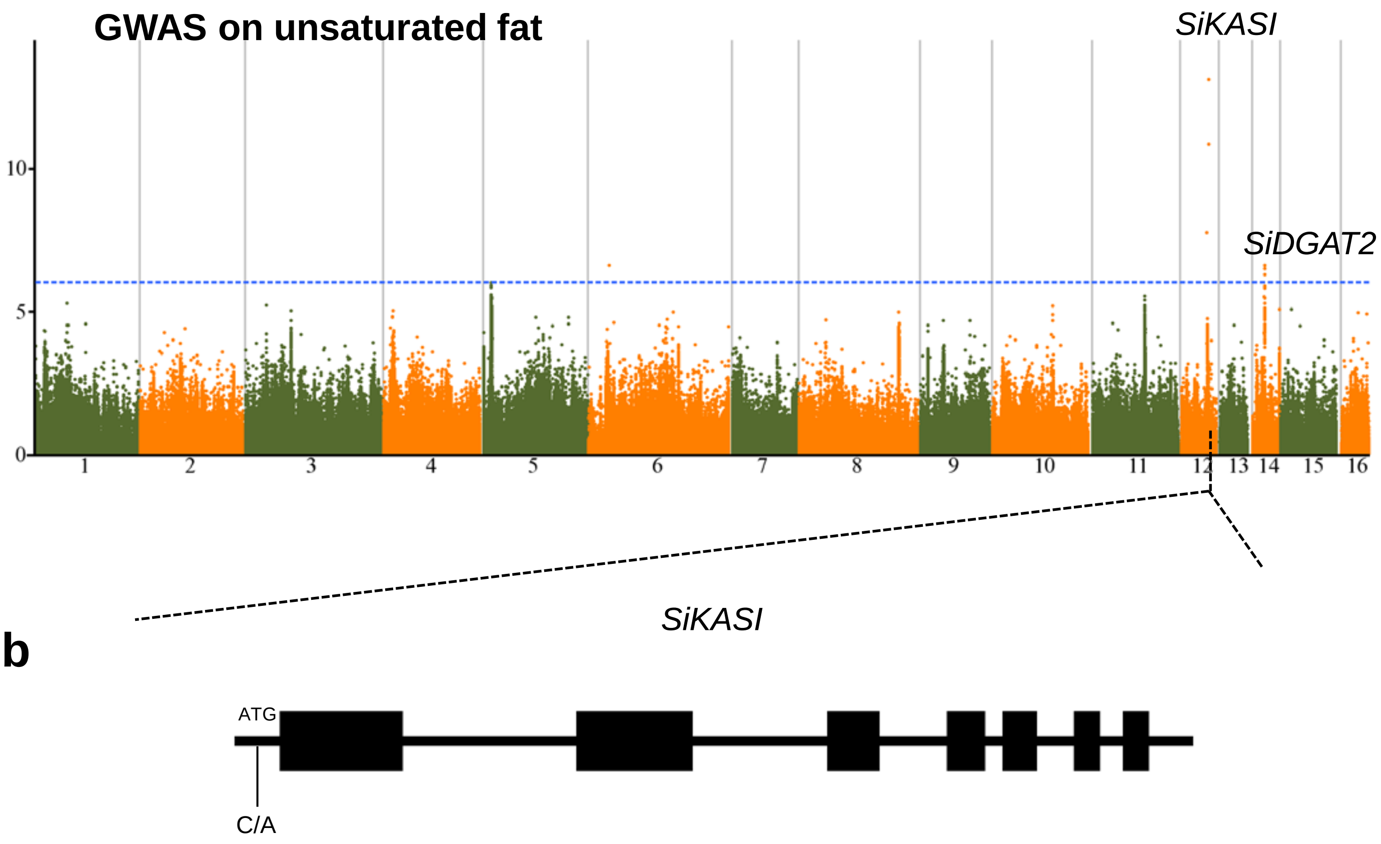

Supplementary Figure 18 Candidate causative gene and variant underlying unsaturated fat. (**a**) Negative $\log_{10}$ *P*-values for association of unsaturated fat (Y-axis) are plotted against SNP positions (X-axis). The genome-wide significant *P* value threshold (10^{-6}) is indicated by a horizontal dash-dot line. The candidate genes are indicated near the association peaks. (**b**) Candidate causative variant in *SiKASI*. The variant in the promoter region was associated with unsaturated fat significantly ($P=8.32\times10^{-14}$).

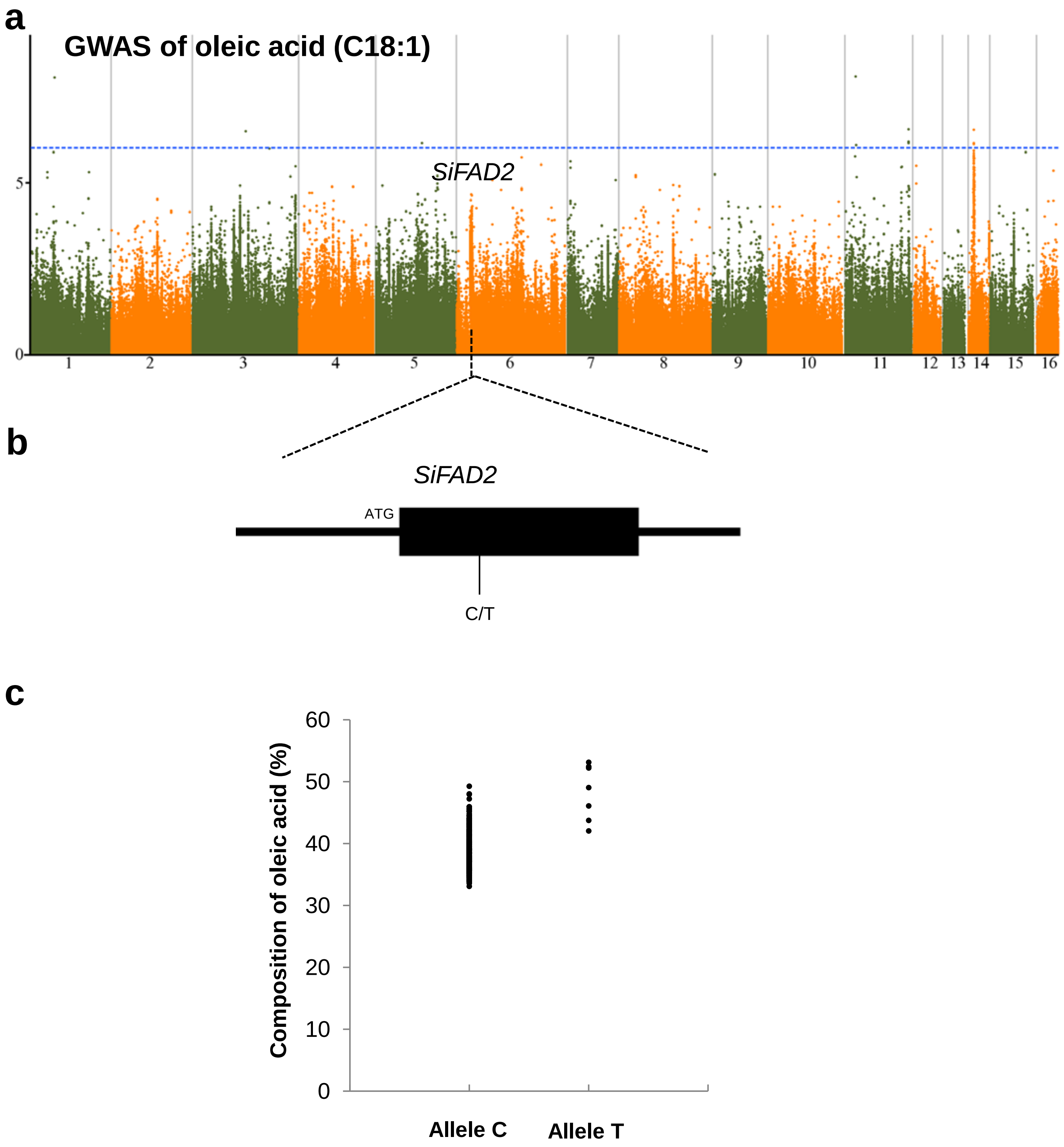

Supplementary Figure 19 Candidate causative gene and variant underlying oleic acid content in sesame seeds. (**a**) Negative $\log_{10}$ *P*-values for association of unsaturated fat (Y-axis) are plotted against SNP positions (X-axis). The genome-wide significant *P* value threshold (10^{-6}) is indicated by a horizontal dash-dot line. (**b**) The candidate causative variant (C/T) in *SiFAD2*. It should be noted that this SNP (C/T) was not included in the GWAS due to low MAF (0.015). (**c**) The varieties with "T" allele show a higher level of oleic acid.

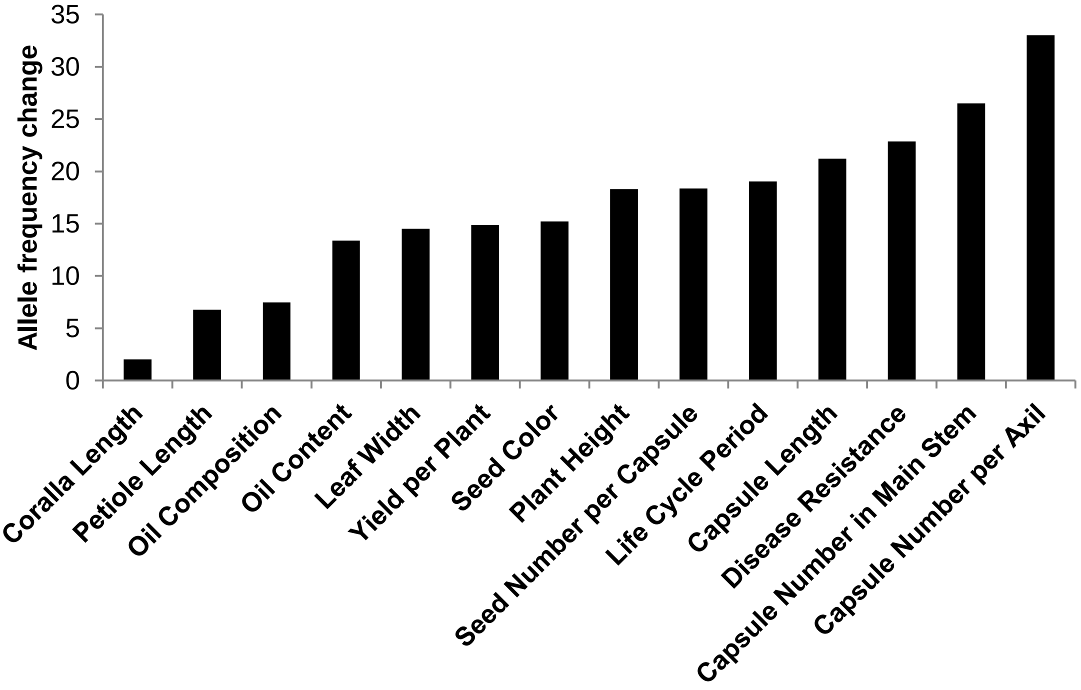

Supplementary Figure 20 Allele frequency changes from landraces to modern cultivars for the lead SNPs underlying different traits. The average is indicated for each trait.

Supplementary Table 1   Summary of whole-genome variants from *de novo* assembly.

	Baizhima	Mishuozhima
**Assembly length**	266,768,502	253,855,660
**Contig N50**	47,222	47,769
**SNPs**	989,930	951,237
**Indels**	362,157	347,359
**Synonymous**	23,986	24,293
**Nonsynonymous**	27,340	27,257
**Large-effect SNPs**	1,037	1,016
**Frameshift indels**	3,211	3,077
**Non-frameshift indels**	8,414	8,095

Supplementary Table 2 The distribution of contigs in two assemblies

Contig size (kb)	Number for the assembly of Baizhima	Total length for the assembly of Baizhima	Number for the assembly of Mishuozhim	Total length for the assembly of Mishuozhim
<1	59529	18,678,728	35590	11,803,433
1-10	8620	26,609,057	8351	26,718,173
10-20	1622	23,660,949	1705	24,924,814
20-30	975	24,130,185	983	24,233,198
30-40	706	24,533,898	670	23,239,064
40-50	482	21,578,994	450	20,109,612
50-60	385	21,035,300	385	21,053,800
60-70	259	16,763,884	271	17,613,725
70-80	192	14,301,124	175	13,125,181
80-90	153	12,964,171	133	11,312,446
90-100	105	9,937,750	123	11,658,029
>100	367	52,574,462	331	48,064,185

Supplementary Table 3 Specificities and missing data rates of the genotype dataset before and after missing genotypes are inferred for common SNPs.

Type	Before imputing	After imputing
The genotype set of 705 sesame accessions (1,805,413 SNPs with MAF > 0.03)		
Specificity (High-coverage sequencing for independent evaluations)*		
G009 19.8x Illumina data	97.6%	97.6%
G010 18.7x Illumina data	97.2%	97.6%
G021 12.4x Illumina data	96.9%	97.4%
G035 18.8x Illumina data	98.2%	97.9%
G145 16.8x Illumina data	98.4%	97.8%
G163 12.6x Illumina data	98.5%	97.8%
G166 16.9x Illumina data	97.3%	97.6%
G174 16.4x Illumina data	98.9%	98.4%
G402 18.2x Illumina data	97.0%	97.8%
G421 13.7x Illumina data	98.6%	98.1%
Missing data rate	47.9%	3.7%

*The genotypic data in GWAS is a large genotype matrix at the 1.8 million SNP sites for each sesame accession. We randomly selected 10 sesame accessions for independent high-coverage sequencing and independent genotype calling at the 1.8 million SNP sites. Assuming the genotype calls from high coverage sequencing data were correct, the specificity of the imputed genotype dataset was calculated for each of the ten sesame accessions.

Supplementary Table 4 Correlation of phenotypic variation for oil content and composition.

	Oil	C16-0	C16-1	C17-0	C17-1	C18-0	C18-1	C18-2	C18-3	C20-0	C20-1	C22-0
**Oil**	1.00											
**C16-0**	-0.14	1.00										
**C16-1**	-0.11	0.76	1.00									
**C17-0**	-0.33	-0.09	-0.05	1.00								
**C17-1**	-0.29	-0.01	0.10	0.61	1.00							
**C18-0**	-0.14	-0.29	-0.34	0.14	-0.02	1.00						
**C18-1**	-0.05	-0.53	-0.36	0.35	0.31	0.16	1.00					
**C18-2**	0.12	0.37	0.25	-0.38	-0.34	-0.24	-0.98	1.00				
**C18-3**	-0.39	0.05	0.01	0.25	0.20	-0.01	0.06	-0.10	1.00			
**C20-0**	-0.28	0.00	-0.01	0.00	-0.11	0.59	0.01	-0.11	0.15	1.00		
**C20-1**	-0.02	-0.26	-0.16	0.14	0.12	-0.14	0.34	-0.29	0.18	-0.05	1.00	
**C22-0**	-0.22	0.09	0.07	0.06	0.03	0.12	-0.03	-0.02	0.12	0.29	-0.07	1.00

Supplementary Table 5 Oilseed yield of the alleles with longer growth stage and larger plant height at four loci.

Candidate gene	Flowering /Height (Allele A)	Flowering /Height (Allele B)	Yield (Allele A)	Yield (Allele B)	*P*-value
**SIN_1021838**	44.76 d	48.29 d	3.03	5.3	2.45E-8
**SIN_1022538**	44.80 d	49.23 d	3.03	6.4	1.24E-9
**SIN_1018135**	103.17 cm	123.15 cm	2.08	3.9	1.38E-6
**SIN_1014512**	101.71 cm	120.23 cm	1.86	3.57	6.94E-6
